# Radiation Dose‐Enhancement Is a Potent Radiotherapeutic Effect of Rare‐Earth Composite Nanoscintillators in Preclinical Models of Glioblastoma

**DOI:** 10.1002/advs.202001675

**Published:** 2020-09-07

**Authors:** Anne‐Laure Bulin, Mans Broekgaarden, Frédéric Chaput, Victor Baisamy, Jan Garrevoet, Benoît Busser, Dennis Brueckner, Antonia Youssef, Jean‐Luc Ravanat, Christophe Dujardin, Vincent Motto‐Ros, Frédéric Lerouge, Sylvain Bohic, Lucie Sancey, Hélène Elleaume

**Affiliations:** ^1^ Synchrotron Radiation for Biomedical Research (STROBE) UA7 INSERM Université Grenoble Alpes Medical Beamline at the European Synchrotron Radiation Facility 71 Avenue des Martyrs Grenoble Cedex 9 38043 France; ^2^ Université de Lyon École Normale Supérieure de Lyon CNRS UMR 5182 Université Claude Bernard Lyon 1 Laboratoire de Chimie Lyon F69342 France; ^3^ Deutsches Elektronen‐Synchrotron DESY Notkestrasse 85 Hamburg DE‐22607 Germany; ^4^ Cancer Targets and Experimental Therapeutics Institute for Advanced Biosciences Université Grenoble Alpes INSERM U1209 CNRS UMR5309 Allée des Alpes La Tronche 38700 France; ^5^ Cancer Clinical Laboratory Grenoble University Hospital Grenoble 38700 France; ^6^ Department Physik Universität Hamburg Luruper Chaussee 149 Hamburg 22761 Germany; ^7^ Université Grenoble Alpes CEA CNRS IRIG SyMMES UMR 5819 Grenoble F‐38000 France; ^8^ Institut Lumière Matière UMR5306 Université Claude Bernard Lyon 1 CNRS Villeurbanne Cedex 69622 France

**Keywords:** glioblastoma, nanoscintillators, radiation dose‐enhancement, synchrotron radiation, X‐ray‐induced photodynamic therapy

## Abstract

To improve the prognosis of glioblastoma, innovative radiotherapy regimens are required to augment the effect of tolerable radiation doses while sparing surrounding tissues. In this context, nanoscintillators are emerging radiotherapeutics that down‐convert X‐rays into photons with energies ranging from UV to near‐infrared. During radiotherapy, these scintillating properties amplify radiation‐induced damage by UV‐C emission or photodynamic effects. Additionally, nanoscintillators that contain high‐Z elements are likely to induce another, currently unexplored effect: radiation dose‐enhancement. This phenomenon stems from a higher photoelectric absorption of orthovoltage X‐rays by high‐Z elements compared to tissues, resulting in increased production of tissue‐damaging photo‐ and Auger electrons. In this study, Geant4 simulations reveal that rare‐earth composite LaF_3_:Ce nanoscintillators effectively generate photo‐ and Auger‐electrons upon orthovoltage X‐rays. 3D spatially resolved X‐ray fluorescence microtomography shows that LaF_3_:Ce highly concentrates in microtumors and enhances radiotherapy in an X‐ray energy‐dependent manner. In an aggressive syngeneic model of orthotopic glioblastoma, intracerebral injection of LaF_3_:Ce is well tolerated and achieves complete tumor remission in 15% of the subjects receiving monochromatic synchrotron radiotherapy. This study provides unequivocal evidence for radiation dose‐enhancement by nanoscintillators, eliciting a prominent radiotherapeutic effect. Altogether, nanoscintillators have invaluable properties for enhancing the focal damage of radiotherapy in glioblastoma and other radioresistant cancers.

## Graphical abstract

Radiation dose‐enhancement induced by rare‐earth composite nanoscintillators is predicted by in silico simulations and unequivocally demonstrates in vitro in microtumor models of glioblastoma, using tunable monochromatic synchrotron radiation. Radiation dose‐enhancement ultimately elicitsa prominent radiotherapeutic effect in a syngeneic orthotopic model of aggressive glioblastoma. These results prove the strong ability of rare‐earth composite nanoscintillators to enhance the focal damage of radiotherapy.

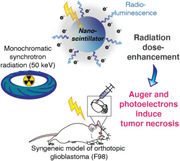

## 1 Introduction

Glioblastoma multiforme is the most common type of primary brain cancer in adults and has a dismal prognosis. The median survival does not exceed one year despite an intensive palliative treatment consisting of a maximal safe surgical resection followed by extensive chemoradiotherapy. The radiation is typically delivered in 2 Gy fractions that, combined, reach a total dose of 60 Gy.^[^ ^1^, ^2^ ^]^ This corresponds to the maximum dose that can safely be delivered to prevent necrosis in healthy brain tissues,^[^ ^3^ ^]^ yet it remains insufficient to adequately control tumor growth. In order to improve the clinical management of glioblastoma by radiotherapy, it is critical to improve the therapeutic window of radiotherapy by increasing the damage done to the tumor without increasing the overall dose delivered to the healthy tissues.

In recent years, a novel therapeutic strategy has emerged to better utilize radiation doses delivered to cancer tissues. This strategy relies on nanoscintillators, which are down‐conversion nanoparticles that absorb X‐rays and emit photons with energies ranging from the ultraviolet to infrared.^[^ ^4^ ^]^ These radioluminescent properties are interesting for biomedical applications,^[^ ^5^, ^6^ ^]^ such as the remote activation of photodynamic therapy (PDT).^[^ ^7^, ^8^ ^]^ Upon X‐ray irradiation, nanoscintillators can transfer energy to nearby/conjugated photosensitizer molecules.^[^ ^9^, ^10^ ^]^ The excited photosensitizers subsequently engage in photochemical reactions that produce high amounts of reactive oxygen species, which can induce severe cytotoxicity in cancer tissues.^[^ ^11^, ^12^ ^]^ Because X‐rays are not limited in terms of tissue penetration compared to visible light, radioluminescence‐activated PDT has the potential to overcome the depth‐limitation of conventional PDT. Recent proof‐of‐concept studies using nanoscintillator‐photosensitizer conjugates demonstrated that this combinatory approach has a strong therapeutic potential in vitro and in vivo.^[^ ^13^, ^14^, ^15^, ^16^, ^17^, ^18^, ^19^, ^20^, ^21^, ^22^, ^23^, ^24^ ^]^ Biomedical applications of nanoscintillators are currently mostly centered on this concept of radioluminescence‐induced PDT. However, factors by which nanoscintillators can augment radiotherapy outcomes also include a potential synergism with radiotherapy^[^ ^25^ ^]^ and the use of nanoscintillators that emit UV‐C photons to expand the amount and types of DNA damage induced during radiotherapy.^[^ ^26^, ^27^, ^28^ ^]^

However, translational studies of nanoscintillators for radiotherapeutic applications are challenged by the lack of biosafety and toxicity profiles. Moreover, the aforementioned proof‐of‐concept studies have almost exclusively been performed on nude mice carrying subcutaneous xenografts, and thus carry limited clinical relevance. One exception is the evaluation of radioluminescence induced‐PDT on an orthotopic lung cancer model.^[^ ^20^ ^]^ This study was the first one to demonstrate the ability of nanoscintillators to induce radioluminescence‐induced PDT in deep‐seated tumor. Most importantly however, remains the uncertainty about the exact therapeutic mechanisms by which augmented radiotherapeutic outcomes are achieved.

It has been demonstrated that the radioluminescence‐yield of nanoscintillators can only enable low‐dose PDT,^[^ ^29^, ^30^, ^31^, ^32^ ^]^ which hints toward the existence of other non‐negligible therapeutic contributions of the nanoscintillators to explain the promising reported efficacies. First and foremost, as nanoscintillators are often composed of high‐Z elements, we hypothesize that a radiation dose‐enhancement effect exists when these materials are used for radiotherapeutic applications. The radiation dose‐enhancement effect occurs as high‐Z elements absorb orthovoltage X‐rays (<250 keV) more efficiently than tissues through photoelectric effect, which generates photo‐ and Auger electrons. These electrons locally enhance the energy deposited in the vicinity of the high‐Z elements,^[^ ^33^, ^34^ ^]^ thereby increasing the extent of DNA damage induced by radiotherapy alone.^[^ ^35^ ^]^ This effect was first reported with iodine‐based contrast agents that showed enhanced cytotoxicity during X‐ray imaging protocols.^[^ ^36^ ^]^ The first in vitro studies performed on iodinated agents demonstrated an important role of the X‐ray energy, as the radiation dose‐enhancement effect only occurred in the orthovoltage range, and is driven by the photoelectric interactions.^[^ ^33^, ^37^, ^38^, ^39^, ^40^, ^41^, ^42^ ^]^ However, as these molecular contrast agents exhibit low tumor retention because of a rapid clearance, high‐Z elements microparticles^[^ ^42^ ^]^ and nanoparticles^[^ ^43^ ^]^ have gained interest. As demonstrated by Monte Carlo simulations, nanoparticles efficiently absorb X‐rays and locally enhance the dose delivered in their close surroundings.^[^ ^29^, ^44^, ^45^ ^]^ A seminal study by Hainfeld et al. reported the prolonged control of subcutaneous tumors by intravenously injecting mice with gold nanoparticles, followed by 250 kVp radiation therapy.^[^ ^46^ ^]^ Many subsequent studies demonstrated physical, chemical, or biological effects of high‐Z nanoparticles upon X‐ray irradiations.^[^ ^43^ ^]^

Recent studies have elucidated that nanoscintillator‐photosensitizer conjugates can realize both radiotherapeutic and photodynamic effects upon X‐ray radiation, and can act as radiosensitizers.^[^ ^47^, ^48^ ^]^ However, whether, and to which extent, nanoscintillators can achieve a radiation dose‐enhancement effect remains uninvestigated. Therefore, prior to undertaking further translational studies toward radioluminescence‐activated PDT and UV‐C radioluminescence induced DNA damage, it is crucial to first determine whether nanoscintillators induce a radiation dose‐enhancement effect and unravel the complete therapeutic mechanisms associated to nanoscintillators and their radiotherapeutic properties. Consequently, this will be critical to optimize the design of nanoconjugates for further studies on radioluminescence‐induced PDT, as well as of nanoscintillators developed to potentiate radiotherapy by UV‐C radioluminescence.

In this paper, we present a comprehensive study of the ability of LaF_3_:Ce nanoscintillators to induce a radiation dose‐enhancement effect. Because their emission does not overlap the absorption of DNA, only the radiation dose‐enhancement is expected. In addition, LaF_3_:Ce nanoscintillators have been previously used to excite, upon X‐ray irradiation, two clinically used photosensitizers,^[^ ^10^, ^16^ ^]^ making it an excellent model material for this study. The radiation dose‐enhancement effect of LaF_3_:Ce was first investigated by custom‐written Geant4‐based Monte Carlo simulations. Subsequently, two different 3D models of glioblastoma were used to study LaF_3_:Ce uptake and localization with state‐of‐the‐art 3D X‐ray fluorescence microtomography, whereas toxicity and radiotherapy efficacies were quantified by multiparametric assessment of the treatment effects on these in vitro models. Second, we evaluated the safety, toxicity, and radiotherapy enhancement in an aggressive syngeneic in vivo model of orthotopic glioblastoma. Throughout the study, radiation therapy was performed with orthovoltage (keV) monochromatic X‐rays delivered by a synchrotron radiation source, which offers the unique opportunity to investigate the underlying mechanisms responsible for the therapeutic efficacy and to unequivocally identify the existence of a dose‐enhancement effect.

## 2 Results and Discussion

### 2.1 Synthesis and Characterization of LaF_3_:Ce Nanoscintillators

LaF_3_:Ce is a luminescent material exhibiting well‐characterized scintillating properties (for single crystal, light yield ≈2200 photons.MeV^−1^; main decay time:≈26 ns).^[^ ^49^ ^]^ When doping the LaF_3_ crystal lattice with Ce, the intermittent La–Ce substitutions are responsible for the radioluminescence emission. The emission band is composed of two peaks (285 and 305 nm), which correspond to the standard 5d (Ce^3+^) → 4f (Ce^3+^) radiative transitions between the lowest excited level of Ce^3+^ and its ground level, split by the spin‐orbit coupling.^[^ ^50^ ^]^ This transition is insufficient to produce photons with energy higher than approximately 280 nm. Therefore, it should be noted that LaF_3_:Ce does not emit in the UV‐C, so radioluminescence‐induced DNA damage by these particles will not occur. In addition, a third peak appears at 340 nm and is attributed to perturbed Ce^3+^ sites, regularly reported in crystals and in nanostructures.^[^ ^51^, ^52^ ^]^ For radiotherapeutic applications, near‐UV emission is advantageous, as these emission peaks overlap with the absorption of chlorin or porphyrin photosensitizers. Therefore, LaF_3_:Ce nanoparticles are ideally suited for conjugation to such photosensitizers and enable radioluminescence‐activated photodynamic therapy.^[^ ^10^, ^16^ ^]^ As both La (Z = 57) and Ce (Z = 58) are high‐Z elements, combined with the lack of UV‐C emission, LaF_3_:Ce particles are ideal candidates to investigate the sole role of a potential radiation dose‐enhancement effect induced during radiotherapy.

**Figure 1:**
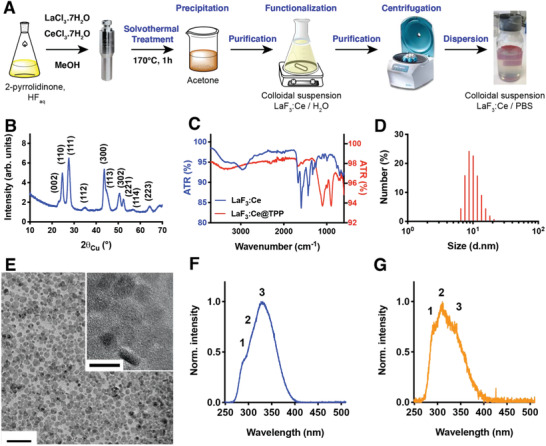
Synthesis and characterization of the LaF_3_:Ce nanoparticles. A) Schematic representation of the chemistry synthesis. B) X‐ray diffractogram of the La_0.9_Ce_0.1_F_3_ nanoparticles – the crystallization phase is identified as being the trigonal tysonite LaF_3_ structure (JCPDS 32‐0483). C) Fourier transform infrared spectroscopy spectra of the LaF_3_:Ce nanoparticles (blue) and the triphosphate‐coated LaF_3_:Ce nanoparticles (LaF_3_:Ce@TPP, red). The attenuated total reflectance (ATR) is plotted as a function of the wavenumber. D) Dynamic light scattering quantifying the hydrodynamic radius of the functionalized nanoparticles. Polydispersity index = 0.12. E) TEM image of the well‐dispersed and well‐crystallized LaF_3_:Ce nanoparticles. Scale bar = 50 nm. Insert: High resolution TEM image. Scale bar = 10 nm. F) Photoluminescence spectrum of LaF_3_:Ce nanoparticles measured with *λ*_exc _= 214 nm. G) Radioluminescence spectrum (30 mA, 33 kV). The peak indexed as 1 corresponds to the transition ^2^D_3/2_^standard^→^2^F_5/2_, the peak indexed as 2 corresponds to ^2^D_3/2_^standard^→^2^F_7/2_ and the peak indexed as 3 corresponds to ^2^D_3/2_^perturbed^→^2^F*_x_*_/2_.

LaF_3_:Ce nanoparticles composed of 10% Ce and 90% La were synthesized using a solvothermal process (**Figure** **1**A). LaF_3_:Ce nanoscintillators were then coated with triphosphate (TPP) to provide stability and dispersibility in aqueous solvents, and thus to render them biocompatible. The X‐ray diffraction pattern (Figure 1B) shows that all the reflections well match the trigonal tysonite LaF_3_ crystal structure (reference JCPDS 32‐0483), with a broadening of the diffraction lines due to the small size of the particles. The TPP coating was confirmed by Fourier transformed infrared attenuated total reflectance spectroscopy (Figure 1C and further detailed in Section S1, Supporting Information). Quantitative elemental analyses by inductively coupled plasma‐optical emission spectroscopy provides a La/Ce ratio of 9.07, confirming the intended composition of the nanoscintillators. The (La + Ce)/P ratio was equal to 1.86, thereby validating the presence of the TPP coating. The size and shape of the core of the nanoparticles were assessed using transmission electron microscopy (TEM), demonstrating that the well‐crystallized and well‐dispersed nanoparticles exhibit a platelet shape of approximately 10 nm wide and about 5 nm thick, for an average diameter of 8 nm (Figure 1E). This was corroborated by dynamic light scattering analysis, which determined the hydrodynamic diameter of the LaF_3_:Ce@TPP nanoparticles at about 10 nm with a low polydispersity index of 0.12 (Figure 1D).

Subsequently, the optical properties of the LaF_3_:Ce nanoscintillators were investigated. Upon 214 nm excitation, we measured the photoluminescence emission of LaF_3_:Ce (Figure 1F). The emission maximum was at 340 nm, which corresponds to the emission of Ce ions located in perturbed sites. The scintillating properties of the LaF_3_:Ce particles were confirmed by radioluminescence measurements, displaying a strong emission upon X‐ray excitation (33 kVp) peaking at 310 nm, which corresponds to the regular emission of Ce (Figure 1G). An elaborate discussion on the difference between the photo‐ and radio‐luminescence spectra is provided in the Section S2, Supporting Information. The characterization of the chemical, photo‐, and radio‐luminescent properties indicate that we successfully synthesized LaF_3_:Ce@TPP nanoscintillators, which will be referred to as LaF_3_:Ce from this point onward.

### 2.2 In Silico Simulations Reveal the Ability of LaF_3_:Ce Nanoscintillators to Induce a Radiation Dose‐Enhancement Effect

**Figure 2:**
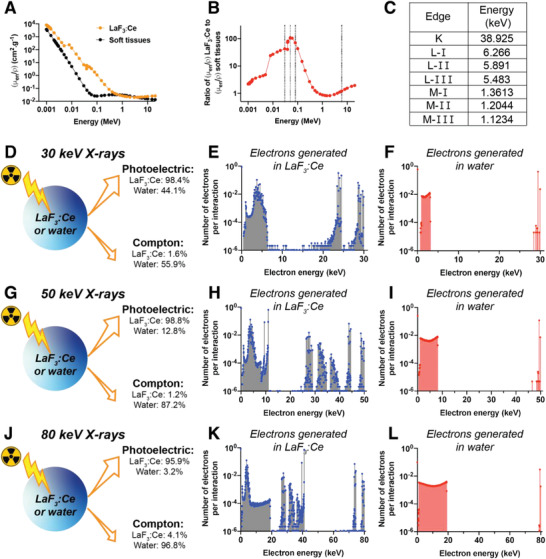
In silico simulations of the interactions between orthovoltage X‐ray photons and LaF_3_:Ce. A) Mass energy absorption coefficients as a function of the X‐ray energy for LaF_3_:Ce and soft tissues. Data obtained from.^[^ ^53^ ^]^ B) Ratio between the mass energy‐absorption coefficient of LaF_3_:Ce and soft tissues. Dashed lines are plotted as eye‐guidance at 30 keV, 50 keV, 80 keV and 6 MeV. C) Energies of the K‐, L‐, and M‐edges for the La. D–L) Simulations of interactions between X‐ray photons and LaF_3_:Ce or water. Panels (D), (E), and (F) are obtained for 30 keV X‐rays, panels (G–I) for 50 keV photons, and panels (J–L) for 80 keV photons. For each X‐ray energy, the percentage of interactions occurring through photoelectric or Compton effects are indicated in panels (D), (G), and (J). (E,F), (H,I), and (K,L) Spectra of Compton, Auger and photo electrons generated after the interaction between an X‐ray photon and LaF_3_:Ce (E,H, and K) or water (F,I, and L).

We first evaluated the theoretical radiation dose‐enhancement expected from pure LaF_3_:Ce relative to soft tissues. **Figure** **2**A represents the mass energy‐absorption coefficient of LaF_3_:Ce and soft tissues as a function of the X‐ray energy.^[^ ^53^ ^]^ The relative absorption of LaF_3_:Ce compared to soft tissues was calculated by dividing these two spectra (Figure 2B). At an energy of 50 keV, the dose deposition in presence of LaF_3_:Ce is 107.2 times higher compared to soft tissue alone. For 30 and 80 keV, this ratio reaches 42.2 and 70.7, respectively. This indicates that an X‐ray energy of 50 keV is likely to induce the highest radiation dose‐enhancement effect. A more detailed explanation is provided is Section S3, Supporting Information. To further investigate the effects of the irradiation energy, we performed Monte Carlo simulations with monochromatic 30, 50, and 80 keV X‐rays, using Geant4.^[^ ^54^ ^]^ When an X‐ray photon interacts with matter, several processes can occur with a probability that is directly related to the photon energy. For orthovoltage X‐rays, photoelectric and Compton interactions are the two effects likely to be observed. The Geant4 simulations demonstrate that photoelectric effect occurs at 98.4%, 98.8%, and 95.9% of the interactions in LaF_3_:Ce for X‐rays of 30, 50, and 80 keV, respectively. In contrast, photoelectric interactions only occur at 44.1%, 12.8%, and 3.2% in water for X‐rays of 30, 50, and 80 keV, respectively (Figure 2D,G,J). The spectra of the electrons generated in LaF_3_:Ce and in water after the primary interactions are represented in Figure 2E,F,H,I,K,L. The electron spectra depict that three types of electrons are generated upon interaction with orthovoltage X‐rays. First, photoelectrons are emitted with an energy spectrum defined by *E*_photoelectron_ = *E*_X‐ray_ − *E*_binding_, for which the binding energies are provided in Figure 2C.^[^ ^55^ ^]^ Second, Auger electrons are generated with energies that depend on the intercrossing mechanisms. Their energies are therefore related to the energies of the K‐, L‐, and M‐shells of the material. Finally, Compton electrons are emitted with an energy that forms a continuous spectrum, as defined by the Compton scattering formula. The spectra of the secondary photons are represented in Figure S1, Supporting Information. These secondary photons are generated by two mechanisms. First, Compton scattering generates photons with a continuous spectrum. Second, X‐ray fluorescence emission occurs, of which the photon energy relates to the energy difference between the involved energy levels.

The radiation dose‐enhancement effect mostly comes from the contribution of the photoelectrons and to a lower extent to the Auger electrons that are extensively produced in LaF_3_:Ce compared to water (i.e., soft tissues). These secondary electrons have energies that, when traveling beyond the boundaries of the nanoparticles,^[^ ^29^ ^]^ will grant them a mean free path of up to a few tens of micrometers in tissues. Because these migration distances exceed the size of a single cell, the radiation dose‐enhancement effect not only affects the cells that incorporated the nanoparticles but may also impact cells located more distal from the nanoparticles. Altogether, these results indicate that the radiation dose‐enhancement effect will be maximized at 50 keV as the ratio between the absorption of the LaF_3_:Ce and soft tissues is highest at this energy. It is also the energy that leads to more photoelectric interactions in LaF_3_:Ce compared to tissues, generating many photoelectrons that will travel up to 100 µm in tissues and create damage in a wide perimeter around the nanoparticles. Moreover, these results indicate that quantifying the biological responses obtained with monochromatic radiotherapy delivered at 30, 50, and 80 keV will provide key evidence on whether LaF_3_:Ce induce a radiation dose‐enhancement effect. These Monte Carlo simulations provide encouraging results to further study the radiation dose‐enhancement effect induced by LaF_3_:Ce nanoparticles in vitro and in vivo.

### 2.3 LaF_3_:Ce Are Well‐Tolerated up to 1 mg mL^−1^ in Glioblastoma Spheroids

The radiation dose‐enhancement effect is most effective with high intratumor concentrations of high‐Z elements,^[^ ^35^ ^]^ making it imperative that the LaF_3_:Ce nanoscintillators are well tolerated at high doses to achieve such concentrations. We first evaluated the toxicity of the LaF_3_:Ce nanoscintillators in glioblastoma spheroids to define the maximum tolerable dose in vitro. 3D culture models of cancer better recapitulate the in vivo features of cancer tissues compared to conventional monolayer cultures,^[^ ^56^ ^]^ yet they remain high‐throughput models to identify the effects of novel treatment regimens.^[^ ^25^, ^57^, ^58^ ^]^ Moreover, 3D microtumor models are more accurate in predicting in vivo radiotherapy outcomes compared to conventional 2D cultures, as we previously discussed.^[^ ^59^ ^]^

**Figure 3:**
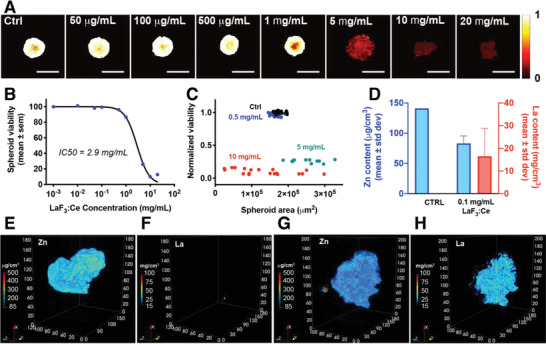
LaF_3_:Ce strongly accumulate and distribute well within F98 models of glioblastoma at non‐toxic concentration. A) Viability heatmaps of F98 spheroids subjected to increasing concentration of LaF_3_:Ce nanoscintillators for 24 h. White pixels indicate a maximal viability, whereas black pixels depict minimal viability. Scale bar = 500 µm. B) Viability of F98 spheroids measured after 24 h of incubation with increasing concentrations of LaF_3_:Ce. Data represents average ± standard errors, *N* = 7–25 spheroids/group from two technical repeats. C) Normalized viability plotted as a function of the spheroid area for selected LaF_3_:Ce concentrations. Each data point represents a single spheroid. D) Quantification of the amount of Zn (physiological element present in the cells) and La contained in the spheroids by analyses of the images acquired by X‐ray fluorescence microtomography. Data represents mean ± standard deviation. E‐H) Images acquired using X‐ray fluorescence microtomography of a spheroid containing no nanoparticle (E,F) and of a spheroid that was exposed to 0.1 mg mL^−1^ LaF_3_:Ce for 24 h. E,G) Zn concentration. F,H) La concentration. The *x*, *y*, and *z* axis are expressed in µm.

Two glioblastoma spheroid models were established from two radioresistant glioblastoma cell lines: rat F98^[^ ^60^, ^61^ ^]^ and human U‐87 MG.^[^ ^62^, ^63^ ^]^ The toxicity of the LaF_3_:Ce nanoscintillators was investigated using dose‐escalation studies by assessing the size and viability of the spheroids after 24 h of exposure. In F98 spheroids, the IC_50_ of the LaF_3_:Ce nanoscintillators was 2.9 ± 0.1 mg mL^−1^ (**Figure** **3**A,B), with no notable decrease in viability observed at concentrations below 1.0 mg mL^−1^ LaF_3_:Ce. When plotting the viability as a function of the spheroid area, a concentration of 5.0 mg mL^−1^ was shown to decrease the spheroid viability down to approximately 20% combined with larger and more heterogeneous spheroid areas (Figure 3C). The latter is indicative of spheroid disruption and loss of cell–cell adhesions. At higher concentrations, the viability is homogeneously decreased and the spheroids are undergoing further disruption, leading to a heterogeneous distribution of spheroid areas within each treatment group. In comparison to spheroids composed of F98 cells, the U‐87 MG spheroids were less sensitive to LaF_3_:Ce toxicity (Figure S2, Supporting Information). Based on spheroid viability, the IC_50_ of the LaF_3_:Ce was 5.2 ± 0.2 mg mL^−1^ (Figure S2B, Supporting Information). When plotting the viability in relation to the spheroid area, it is shown that the viability of U‐87 MG spheroids exposed to 5.0 mg mL^−1^ is homogenously decreased to 50%, while their size remains widely distributed. However, for higher concentrations, the viability is reduced to ≤20% and the spheroid area also strongly and homogeneously decreases (Figure S2C, Supporting Information).

Taken together, our results indicate no acute toxicity of concentrations up to 1.0 mg mL^−1^ of LaF_3_:Ce nanoscintillators in both glioblastoma spheroid types. Further experiments were performed with a concentration of 0.1 mg mL^−1^, to ensure a non‐toxic regimen.

### 2.4 LaF_3_:Ce Nanoparticles Strongly Accumulate and Distribute Well within Glioblastoma Spheroids

To investigate the uptake and distribution of LaF_3_:Ce nanoscintillators in glioblastoma spheroids, we performed 3D‐resolved X‐ray fluorescence microtomography. Whereas this technique has been used for 2D elemental imaging in spheroids,^[^ ^64^ ^]^ this study is the first to report the use of this innovative microtomography method for 3D/spatially resolved elemental imaging on spheroids. Quantitative 3D‐maps were reconstructed for glioblastoma spheroids that were previously incubated with 0.1 mg mL^−1^ LaF_3_:Ce nanoparticles for 24 h prior to imaging (Figure 3E–H and Figure S2E–H, Supporting Information, for F98 and U‐87 MG, respectively). Zinc is a physiological element distributed within the cells and more particularly throughout the nuclear region, thus enabling the delineation of the shape and volume of the multicellular tumor spheroids.

The concentration of La was measured throughout the entire spheroid to localize the nanoscintillators. Microtomography imaging revealed that the nanoparticles accumulate more in the U‐87 MG spheroids than in the F98 spheroids as the average concentrations of LaF_3_:Ce reached approximately 16.0 mg mL^−1^ versus 25.0 mg mL^−1^ for F98 and U‐87 MG, respectively (Figure 3D; Figure S2D, Supporting Information). This data corresponds to a 160‐ and 250‐fold concentration of the initial 0.1 mg mL^−1^ suspension of LaF_3_:Ce nanoparticles within the spheroids. The differences observed between the two cell lines may be explained by the morphological differences between the two types of spheroids: the F98 spheroids are more compact compared to U‐87 MG spheroids, which may have limited the diffusion of the nanoparticles. However, in both cases, the accumulation of LaF_3_:Ce nanoparticles reaches values that largely exceed 1 mg mL^−1^, which is typically estimated as the minimal concentration necessary to induce a significant dose‐enhancement effect.^[^ ^35^ ^]^

### 2.5 LaF_3_:Ce Nanoscintillators Enhance Radiotherapy Efficacy In Vitro through a Radiation Dose‐Enhancement Effect

**Figure 4:**
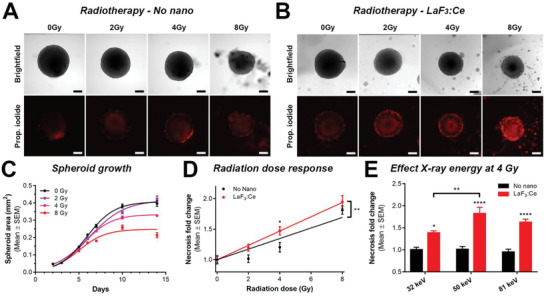
LaF_3_:Ce nanoscintillators induce a radiation dose‐enhancement effect in vitro on F98 spheroids. A,B) Confocal fluorescence microscopy images of F98 spheroids taken on culture day 10, that is, 7 days following radiotherapy delivered in absence of nanoscintillators (A), or after a 24 h incubation with 0.1 mg mL^−1^ LaF_3_:Ce (B). Representative brightfield and propidium iodide emission (necrosis) images of each treatment conditions are shown. Scale bar  =  100 µm. C) Logistic growth curves of F98 spheroids treated with synchrotron radiation therapy (no nanoparticle) at 50 keV with a dose of 0 Gy (black), 2 Gy (purple), 4 Gy (pink), or 8 Gy (red). D) Radiotherapy dose‐response fitted as a function of spheroid necrosis. Data was normalized to the 0 Gy controls of each group, and fitted with linear regression. The slope was significantly different (*p* = 0.036). At each dose, the data from both groups was statistically different compared with a one‐way ANOVA and Sidak's multiple comparisons test. E) Analysis of spheroid necrosis following radiotherapy at photon energies of either 30, 50, or 80 keV. Data was pooled from various time‐points, and plotted as a fold‐change compared to the “No nano” control groups subjected to the same radiotherapy dose. Data was statistically analyzed with a with a one‐way ANOVA and Sidak's multiple comparisons test. In all panels, statistical significance is indicated as “*”(*p* < 0.05), “**”(*p* < 0.01), “***”(*p* < 0.005), or “****”(*p* < 0.001).

We next investigated the capacity of the LaF_3_:Ce nanoscintillators to augment radiotherapy efficacy in vitro. F98 and U‐87 MG glioblastoma spheroids were established and incubated for 24 h with 0.1 mg mL^−1^ LaF_3_:Ce nanoscintillators; these parameters previously allowed us to achieve sufficient intracellular LaF_3_:Ce concentrations to enable a potential radiation dose‐enhancement effect, while being non‐toxic for spheroids (Figure 3; Figure S2, Supporting Information). After incubation, the spheroids were washed to remove unbound LaF_3_:Ce, and were immediately treated with monochromatic synchrotron radiotherapy. In F98 spheroids, 50 keV monochromatic synchrotron radiation induced a dose‐dependent growth inhibition (**Figure** **4**A–C), which was minimally influenced by the presence of LaF_3_:Ce. As the treatment effects appeared to be static from day 10 onwards, this time point was selected to further investigate the state of the spheroid cultures. A dose‐dependent increase in F98 spheroid necrosis was observed, which was significantly higher in spheroids containing LaF_3_:Ce (Figure 4B,D). To verify whether this increase is related to a radiation dose‐enhancement effect induced by the presence of high‐Z elements in the spheroids, we separately irradiated the spheroids with 4 Gy monochromatic synchrotron radiation at either 30, 50, and 80 keV, which correspond to energies below, centered, and above the maximum absorption difference with soft tissues (Figure 2B).^[^ ^29^, ^53^ ^]^ Although there were significant increases in spheroid necrosis in presence of LaF_3_:Ce at 30 and 80 keV, relative to their control groups, the highest elevation in spheroid necrosis was observed at 50 keV (Figure 4E).

In U‐87 MG spheroids, a similar radiation dose‐dependent inhibition of spheroid growth was observed following 50 keV monochromatic synchrotron radiation (Figure S3, Supporting Information). We additionally observed that in the presence of LaF_3_:Ce, there was a significant elevation in spheroid necrosis (Figure S3D, Supporting Information) and reduction in spheroid sizes (Figure S3E, Supporting Information), in comparison to radiotherapy alone. By irradiating the U‐87 MG spheroids with 4 Gy delivered at either 30, 50, or 80 keV (Figure S3F–H, Supporting Information), a radiation dose‐enhancement effect by LaF_3_:Ce was observed, as well as an elevation in spheroid necrosis (Figure S3G, Supporting Information) and a reduction in spheroid area (Figure S3H, Supporting Information). Taken together, our findings indicate that LaF_3_:Ce nanoscintillators can significantly amplify radiotherapy efficacies in glioblastoma spheroids derived from two distinct radioresistant cell lines. Furthermore, the results indicate that these effects can be attributed, in part, to a radiation dose‐enhancement effect. Supported by our simulations (Figure 2), high‐Z elements increase the photoelectric absorption of the incoming X‐rays, which leads to an over‐production of photo‐and Auger electrons that increase the extent of cell damage, culminating in higher degrees of necrosis.

Altogether, these findings provide compelling evidence for the potential of LaF_3_:Ce nanoscintillators to enhance the efficacy of radiation therapy through a radiation dose‐enhancement effect, encouraging further in vivo investigations.

### 2.6 LaF_3_:Ce Nanoscintillators Can Be Safely Administered to Healthy Rats

To identify maximum tolerable doses of LaF_3_:Ce nanoscintillators in vivo, we performed dose‐escalation studies for two clinically relevant injection routes in healthy rats. Intravenous (iv) injection was selected as it is easily implemented experimentally and has translational value. However, it remains challenging to accumulate large amounts of nanoparticles in the brain via this administration route because of the blood–brain barrier, despite that it may be partially disrupted in the presence of brain tumors.^[^ ^65^ ^]^ Therefore, we additionally evaluated the intracerebral delivery of the nanoparticles through a convection‐enhanced delivery (CED) injection. Intracerebral CED refers to a slow injection of the compound directly in the tumor mass by creating a pressure gradient at the tip of the injection syringe. This pressure is created by an infusion pump connected to the syringe.^[^ ^66^ ^]^ This technique is clinically investigated and is a valuable alternative to deliver therapeutics directly into the brain and circumvent the blood‐brain barrier.^[^ ^66^ ^]^ In our protocol, the intracerebral CED injection was performed in the right caudate nucleus, at similar stereotactic coordinates where tumors will be implanted for radiotherapy experiments.

**Figure 5:**
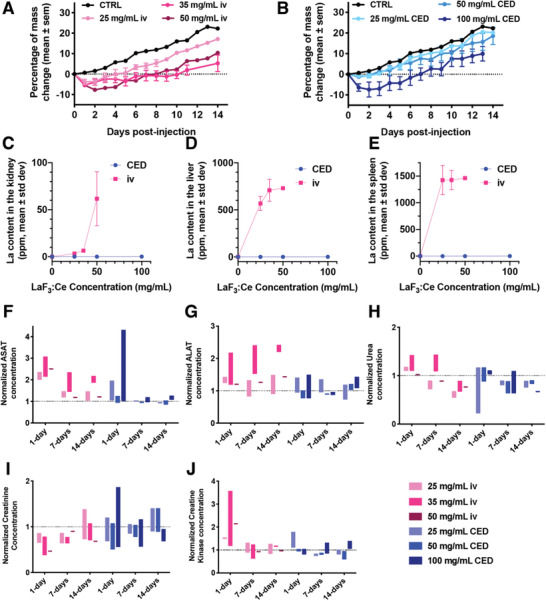
Toxicology investigation indicates that 35 and 50 mg mL^−1^ of LaF_3_:Ce can safely be injected by intravenous (iv) and CED, respectively. A,B) Growth curves of the animals that received LaF_3_:Ce nanoparticles by iv and CED injections, respectively. C–E) Concentration of La measured in the kidney, liver, and spleen, respectively, for increasing concentrations of LaF_3_:Ce injected by iv (pink) and CED (blue). Data represents mean ± standard deviation, *N* = three/groups. F–J) Concentration of aspartate transaminase (ASAT), alanine transaminase (ALAT), urea, creatinine, and creatine kinase, normalized on the values obtained for the control group, measured after LaF_3_:Ce injection by iv (pink) and CED (blue). The data show a bar ranging from the lowest to the highest value, *N* = 3 animals/group.

Our findings are detailed in **Figure** **5**, Figure S4, Supporting Information, and in Section S6, Supporting Information. In summary, CED injection of LaF_3_:Ce was safe and well‐tolerated up to 9.6 mg kg^−1^ body weight (bw), with no noticeable retention in the liver, kidney, or spleen. As for iv injection, LaF_3_:Ce nanoscintillators were lethal at concentrations exceeding 168 mg kg^−1^ bw. Although LaF_3_:Ce was retained in the kidneys, liver, and spleen following iv injection, no long‐term toxicity was observed. These results are encouraging to further investigate the biodistribution of LaF_3_:Ce in rat bearing glioblastoma.

### 2.7 Determination of the Optimal Injection‐to‐Irradiation Delay and Kinetics of Elimination

**Figure 6:**
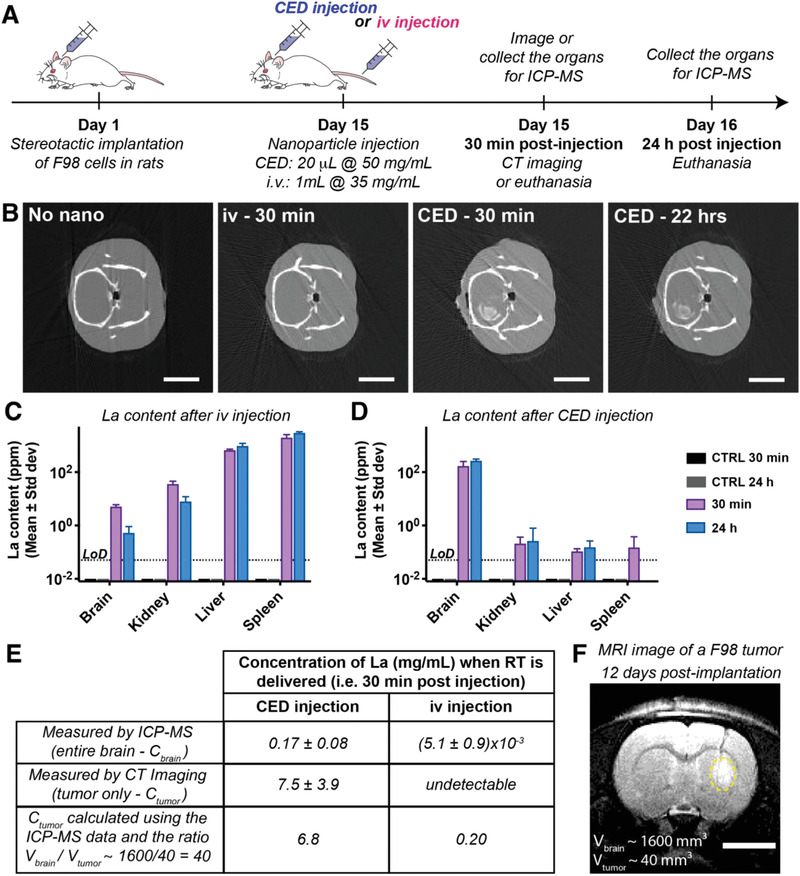
30 min is an optimal injection‐to‐irradiation delay. A) Experiment timeline: on day 1, F98 cells are implanted in Fischer rats. Fourteen days post‐implantation, the nanoparticles are injected by intravenous (iv) or CED. 30 min or 24 h after injection, animals are euthanized and their organs are collected for inductively coupled plasma mass spectrometry (ICP‐MS) analysis. In addition, computed tomography (CT) images are acquired 30 min and about 24 h post‐injection. B) Representative CT images of a control animal (no nanoparticles), as well as three animals that received an iv injection 30 min earlier, or a CED injection 30 min or 22 h earlier. Scale bar = 10 mm. C,D) La measured in each organ after iv (C), or CED injection (D). The caption is identical for both graphs. The data shows mean ± standard deviation. *N* = 4/group. LoD: limit of detection of the ICP‐MS system. E) La measured in the brain 30 min post‐injection by ICP‐MS and CT. The La concentration in the tumor was extrapolated from the ICP‐MS measurements (whole brain) and estimated at 6.8 mg mL^−1^ after CED, comparable to the value of 7.5 mg mL^−1^ measured by CT imaging. A similar extrapolation indicated a concentration 0.2 mg mL^−1^ La in the tumor 30 min post iv injection. F) Representative T2‐sequence magnetic resonance imaging (MRI) image of a rat bearing a F98‐tumor 12 days after tumor inoculation. Scale bar = 5 mm. Tumor tissue is delineated with a yellow dashed line.

Upon determining safe doses for intracerebral CED and iv administration, we next investigated the tumor accumulation and retention of LaF_3_:Ce in vivo. We chose a syngeneic rat model of orthotopic glioblastoma: the F98 model. LaF_3_:Ce were injected 14 days after F98‐tumor inoculation in Fischer rats, when tumors typically reach approximately 3 mm in diameter (**Figure** **6**A).^[^ ^67^ ^]^

Our findings are detailed in Figure 6; Figure S5, Supporting Information, and elaborately discussed in Section S7, Supporting Information. In summary, the results indicate that a 30 min injection‐to‐irradiation delay achieves the highest tumor accumulation of LaF_3_:Ce for both CED and iv injection. We can additionally hypothesize that a potential radiation dose‐enhancement effect is most likely to be achieved with CED injections, as concentrations are sufficiently high in the range of 6.8–7.5 mg mL^−1^. The nanoparticles remain in the brain for a prolonged period of time, which is interesting for a clinical translation, as the total radiation dose given to glioblastoma patients is usually fractionated. Therefore, a single injection of nanoparticles through CED could potentiate several fractions of radiotherapy given over an extended period of time (days‐weeks). In contrast, LaF_3_:Ce tumor concentrations following iv injections reached only 0.2 mg mL^−1^, which is unlikely to be sufficient to achieve a radiation dose‐enhancement effect.

### 2.8 LaF_3_:Ce‐Assisted Radiotherapy Improves Long‐Term Survival in an Aggressive Syngeneic Rat Model of Orthotopic Glioblastoma

**Figure 7:**
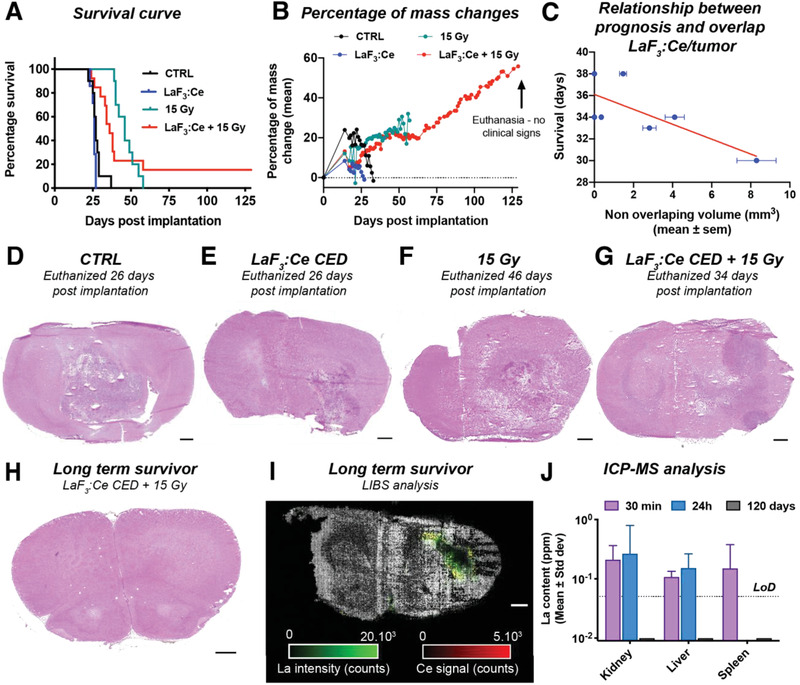
Radiation dose‐enhancement by LaF_3_:Ce leads to a full recovery of 15% of the animals bearing a F98‐brain tumor. A) Survival curve obtained after treatment when LaF_3_:Ce nanoparticles are delivered by CED (20 µL of 50 mg mL^−1^). B) Growth curve of the animals, presented as a percentage of change compared to the mass of the animal on the day of tumor implantation. The two long‐term survivors were euthanized on day 129 (115 days after treatment), although they were showing no clinical signs of pathology. C) Correlation between the survival and the volume of tumor not overlapped by the distribution of nanoparticles. The two long‐term survivors do not appear on this graph as they were euthanized without clinical signs. D–G) Hematoxylin and eosin (H&E) staining of brain slices collected after euthanasia of a representative animal from the control group (D), the group receiving a CED injection only (E), the group receiving only the irradiation (F) and the group receiving the CED injection and the irradiation (G). For this last group, we chose an animal that died early (34 days post tumor inoculation). Scale bar = 1 mm. H) H&E staining of a slice of the brain of one of the two long‐term survivors. Scale bar = 1 mm. I) LIBS image of a consecutive slice of the brain of a long‐term survivor. LaF_3_:Ce nanoparticles are still visible in the brain 129 days post tumor implantation. Scale bar = 1 mm. J) La measured by ICP‐MS contained in the kidney, liver, and spleen of animals euthanized 30 min and 24 h post‐injection (data shown in Figure 6), together with the results obtained for the two long‐term survivor animals. 129 days post implantation (115 days after treatment), no signal was detected in any of these organs, hinting to a total elimination of the La present in the rest of the organism.

We next investigated whether LaF_3_:Ce could improve radiotherapy outcomes in vivo. The F98 model is highly appropriate as it involves animals with an intact immune system. It also recapitulates several important aspects of human glioblastoma such as its high invasiveness, its low immunogenicity, and its resistance to many therapeutic modalities including radiation therapy.^[^ ^61^ ^]^ This model is highly aggressive and was shown to systematically and reproducibly cause the death of the animals within 36 days after implantation of as few as 10 cells in the brain.^[^ ^68^ ^]^ Therefore, an improvement, even minimal, on such an aggressive and clinically relevant model would bring hope for improving glioblastoma outcomes in patients. Regarding the treatment, the previously optimized parameters were applied: a 50 keV monochromatic beam to deliver the radiation, 30 min injection‐to‐irradiation delay for both the iv injection (150 mg kg^−1^ bw) and CED (4.3 mg kg^−1^ bw). A single fraction of 15 Gy was delivered, as we previously demonstrated that this dose increases the median survival by 77%, without curing the animals. In addition, a single fraction of 15 Gy is equivalent for healthy brain tissue to 60 Gy delivered in 30 fractions of 2 Gy,^[^ ^69^ ^]^ which is the dose scheme conventionally used in clinic for glioblastoma.^[^ ^70^ ^]^ Subjects that received no treatment uniformly reached a humane endpoint between 22‐ and 37‐days post‐implantation, showing a mean survival of 27.6 ± 3.9 days (**Figure** **7**A; Figure S7A, Supporting Information, black curve).

With respect to iv administration of LaF_3_:Ce, no clear treatment benefit was observed in this aggressive model. Subjects receiving iv LaF_3_:Ce had a similar overall survival (29.0 ± 2.5 days, Figure S7A, Supporting Information, blue curve), as the control group. The combination of LaF_3_:Ce with 15 Gy radiotherapy achieved no survival benefit (43.8 ± 5.9 days), compared to radiotherapy alone (46.6 ± 6.6 days) (Figure S7A, Supporting Information) and induced a slight, yet non‐significant decreased in the survival. A mild toxicity associated to the use the nanoscintillators combined with radiotherapy in these diseased animals may have contributed to the observed treatment effects, as discussed more elaborately in Section S10, Supporting Information. These findings suggest that approaches toward opening the blood–brain barrier are required to improve the accumulation of iv‐injected nanoparticles in glioblastoma tissues.

For the intracerebral CED injection of LaF_3_:Ce nanoscintillators, the survival uniformly averaged at 25.7 ± 1.4 days post‐implantation (Figure 7A, blue curve), which was not significantly different compared to the no‐treatment group. The treatment outcomes of 15 Gy radiotherapy in subjects receiving intracerebral CED injection of LaF_3_:Ce nanoscintillators was much more irregular compared to 15 Gy radiotherapy alone. Although many animals performed worse in comparison to the group receiving radiotherapy alone, it was observed that 15% of the animals exhibited long term survival (Figure 7A, red curve). The weight of the long‐term survivors evolved normally, and animals were sacrificed 129 days post‐implantation without any signs of disease or discomfort (Figure 7B). Histological analysis confirmed that the brains of the long‐term survivors were tumor‐free upon euthanasia (Figure 7H). In contrast, animals that died at early time points (26 days post‐implantation for the control and CED only groups and 46 and 34 days for the radiotherapy and CED + radiotherapy group, respectively) carried large tumors at autopsy (Figure 7D–G). Elemental analysis with laser induced breakdown spectroscopy (LIBS) qualitatively shows the presence of both La and Ce in the autopsied brain of long‐term survivor (115 days post‐injection) (Figure 7I), as well as in the brain tissues of all CED‐treated animals (Figure S6, Supporting Information). The two long‐term survivors showed no measurable amount of La in the kidney, liver or spleen (Figure 7J), which indicates that the low amounts of La present in these organs at 30 min and 24 h after CED were eliminated within 115 days.

The presence of long‐term survivors following CED + 15 Gy was highly encouraging given the reproducible and uniform behavior of this aggressive model throughout this study and as reported previously.^[^ ^68^ ^]^

### 2.9 Improved Survival Following Radiotherapy Correlates with the Degree of Overlap between LaF_3_:Ce Localization and Tumor Volumes

These promising findings regarding LaF_3_:Ce radiation dose‐enhancement prompted us to further investigate the causes of pre‐emptive mortality in this treatment group. By matching the tumor volumes outlined on magnetic resonance imaging (MRI) images acquired on day 12 with the distribution of the nanoparticles on the computed tomography (CT) images acquired on day 14, we discovered a negative correlation between the survival and the volume of tumor not covered by the nanoscintillators (Figure 7C). Thus, when the distribution of nanoparticles does not entirely cover the tumor volume, part of the tumor mass and the infiltrating cells that are often identified as a cause of relapse will not be properly treated, which negatively affects the survival. Contrariwise, the nanoparticles distributed outside of the tumor volume may also have a harmful effect by inducing an inadequate delineation of the treated area leading to healthy tissue destruction. A comparable observation was made for PDT by Rocha et al., who reported cancer recurrence and a poor improvement of the survival if the tumor irradiation was performed with too narrow margins. Less intuitively, when too wide tumor margins were irradiated, early deaths were reported due to healthy tissue damage. Only after careful optimization of the treated tumor margins, a significant long‐term survival benefit was observed.^[^ ^71^ ^]^

Our findings provided compelling proof‐of‐concept that LaF_3_:Ce nanoscintillators can induce radiation dose‐enhancement in vivo. When administered by intracerebral CED, LaF_3_:Ce nanoscintillators could augment radiotherapy efficacy in vivo, achieving complete tumor remission and long‐term survival in 15% of the subjects. These findings are highly encouraging given the aggressive nature of the F98 tumors and its resemblance to the human pathology.

To further increase the survival rates with LaF_3_:Ce‐enhanced radiotherapy, the nanoscintillators are required to completely cover the tumor volume with high specificity. Whereas intracerebral CED can achieve elevated concentrations of the high‐Z element nanoparticles, it is not ideally suited to achieve a perfect tumor overlap, as observed here and reported previously.^[^ ^72^ ^]^ Although intravenous administration of the LaF_3_:Ce nanoscintillators may be better suited to achieve specific tumor accumulation, new strategies are required to elevate the permeability of the blood‐brain barrier at the cancer site. Recently, an innovative approach using ultrasound was clinically demonstrated successful at temporary opening the blood‐brain barrier to improve the accumulation of intravenously injected chemotherapeutics at the tumor site.^[^ ^73^ ^]^ When combined with such technical innovations, high‐Z element nanoscintillators may prove to be highly valuable for enhancing the focal damage of radiotherapy in glioblastoma and other radioresistant cancers.

### 2.10 Radiation Dose‐Enhancement by Nanoscintillators: Implications for the Design of Nanoconjugates for Radioluminescence‐Activated PDT and Nanoscintillators for UV‐C Induced DNA Damage

The findings of this study have various implications for the development of new nanoscintillators and their biomedical applications. First, as radiation dose‐enhancement is a purely physical effect that is related to the presence of high‐Z elements in materials, it can be simulated in silico, and the radiotherapeutic effects can thus be estimated. Such investigations can be used to optimize the elemental composition of novel nanoscintillators. For example, whereas LaF_3_:Ce was chosen as a model material for this study as it is able to excite several clinically used photosensitizers, nanoscintillators composed of higher Z‐elements may provide a stronger radiation dose‐enhancement effect.^[^ ^29^ ^]^ Second, our findings reveal that the radiation dose‐enhancement effect induced by rare earth composite nanoscintillators is a significant radiotherapeutic mechanism. When tailoring new nanoscintillator‐photosensitizer conjugates for radioluminescence‐induced PDT, trade‐offs may need to be made between the radiation dose‐enhancement effect, the nanoscintillator light‐yield, and the efficacy of the energy transfer occurring between the nanoscintillator and the photosensitizer.^[^ ^9^ ^]^ Third, disease‐specific nanoscintillator‐photosensitizer nanoconjugates may be developed based on the genotypical and phenotypical sensitivity of the target cancer type to X‐ray radiation‐induced DNA damage, UV‐induced DNA damage, and photodynamic therapy.

Last, this study compared the iv administration to CED in terms of toxicity, tumor uptake, and radiotherapy efficacy of the nanoscintillators. Our findings reveal that for glioblastoma, the CED achieves intratumor concentrations that enable a radiation dose‐enhancement effect, whereas iv administration of nanoscintillators did not. Although efforts to better cover and confine the distribution of the nanoscintillators to the tumor volume are necessary, this administration route holds promise in the development of radioluminescence‐activated PDT for glioblastoma. These findings suggest that approaches toward opening the blood‐brain barrier are required to improve the accumulation of iv‐injected nanoparticles in glioblastoma tissues.

## 3 Conclusion

In conclusion, our findings elucidate three critical points regarding the use of rare‐earth composite nanoscintillators for radiation therapy. First, we discovered that rare‐earth composite nanoscintillators can induce a potent radiation dose‐enhancement effect, capable of improving radiotherapy outcomes in an aggressive preclinical model of glioblastoma. Further improvement of the selectivity and specificity of the nanoscintillators for tumor cells is crucial to eliminate the treatment‐induced lethality and maximize the therapeutic benefit in a highly focal manner. Second, this radiation dose‐enhancement effect needs to be considered when designing and evaluating novel scintillating nanoparticles for radiotherapeutic applications. The therapeutic contributions of these photochemical and physical radiotherapeutic effects may be comparable and potentially mechanistically synergistic. Finally, our findings also point out the critical need of using < 250 keV, orthovoltage X‐rays when investigating radiotherapeutic applications of nanoscintillators, as both the radiation dose‐enhancement effect and the photochemical effects will benefit from low energy X‐rays.^[^ ^29^, ^74^ ^]^ Synchrotron radiation has been safely used in a recent clinical trial to deliver 80 keV monochromatic radiation for the treatment of humans with brain tumors.^[^ ^75^ ^]^ Alternatively, brachytherapy can be considered for the internal delivery of kilovoltage X‐rays. These emerging technical and clinical innovations illuminate a bright future for radiotherapeutic contributions of rare‐earth composite nanoscintillators in cancer therapy.

## 4 Experimental Section

##### Synthesis of Aqueous Colloidal Suspensions of Cerium Doped LaF_3_ Nanoparticles (La_0.9_Ce_0.1_F_3_)

In a typical synthesis, 10.36 g (27,9 mmol) of lanthanum (III) chloride heptahydrate (LaCl_3_.7H_2_O, Aldrich) and 1.16 g (3.1 mmol) of cerium (III) chloride heptahydrate (CeCl_3_.7H_2_O, Aldrich) were dissolved in 23 mL of methanol (solution 1). A second solution was prepared by mixing 2.82 g (69.7 mmol) of hydrofluoric acid (49.5 wt% in H_2_O, Aldrich) with 124 mL of 2‐pyrrolidinone (solution 2). Thereafter, solution 1 was quickly added to solution 2 while stirring with the aid of a magnetic stirrer. After stirring for a few minutes, a light‐yellow transparent solution was obtained. The reaction medium was then transferred into a Teflon‐lined stainless‐steel autoclave (Berghof, DAB‐3, inner volume: approximately 210 mL). The pressure vessel was sealed, heated to 170 °C, and held at that temperature for a period of 1 h while keeping the reaction medium under stirring. After cooling down, the reaction crude obtained was introduced into approximately 160 mL of acetone. The precipitation of La_0.9_Ce_0.1_F_3_ nanoparticles was immediate and complete in the form of brownish flakes. These were isolated by centrifugation. The supernatant was carefully removed and the brown pellet was resuspended in methanol with the aid of ultrasound and then centrifuged. This washing process was repeated twice. Finally, the brown precipitate was dispersed in deionized water so as to obtain a slightly brown transparent colloidal solution with a dry extract of 10%.

The functionalization of the nanoparticles with phosphate molecules was carried out using the colloidal solution prepared in the previous step. A solution of pentabasic sodium triphosphate (2.31 g in 25 mL of deionized water) was added to the solution of nanoparticles under very vigorous stirring. The initially transparent solution quickly clouded with an increase in its viscosity. The resulting solution was stirred overnight at room temperature. The purification of the functionalized nanoparticles was carried out by precipitation in ethanol and centrifugation, repeated twice. This step made it possible to get rid of the surface molecules resulting from the degradation of the solvent. Finally, the resulting, very slightly colored centrifuging pellet was dispersed in phosphate buffered saline (PBS), with a final solid content of 1.54%.

##### Nanoparticle Characterizations

TEM was performed on a JEOL JEM 2100 system operating at an acceleration voltage of 200 kV. Diluted nanoparticle suspensions were placed onto carbon‐coated copper grids and dried at room temperature. X‐ray powder diffraction pattern was recorded in the range of 10° ≤ 2*q* ≤ 70° using an Empyrean diffractometer (Malvern Panalytical; CuKα radiation). Fourier transform infrared analysis of the samples was performed using a PerkinElmer spectrophotometer (Spectrum 65 FTIR) equipped with an attenuated total reflectance sample chamber. The hydrodynamic diameter of the nanoparticles in suspension was measured using a Zetasizer Nano ZS (Malvern Panalytical). Chemical component analysis of nanoparticles was performed by inductively coupled plasma‐optical emission spectrometry technique (ACTIVA, Horiba Jobin Yvon). This technique has also been used to calculate the concentration of the nanoparticles in suspension.

##### Photoluminescence and Radioluminescence Measurements

The photoluminescence spectrum was measured upon a 214 nm excitation delivered by a pulsed laser‐driven light source filtered by a Jobin Yvon Gemini 180 monochromator. The light emitted from the sample was collected by an optical fiber connected to a Jobin‐Yvon TRIAX320 monochromator (300 lines.mm^−1^ grating blazed at 250 nm) equipped with a cooled CCD camera. At the entrance of the monochromator, a band‐pass filter (240–395 nm) was installed to remove the excitation light. The radioluminescence spectrum was measured upon X‐ray excitation delivered by an X‐ray generator producing Bremsstrahlung X‐rays (Inel XRG 3000) from accelerated electrons (30 mA‐33 kV) bombarding a tungsten anode. The scintillation spectrum was acquired using an Andor Newton EM‐CCD camera (DU970P‐UVB) coupled to a SR500i‐D2 monochromator (Andor, 149 lines.mm^‐1^ grating blazed at 300 nm). The radioluminescence spectra was measured using a polychromatic X‐ray source. However, no difference is expected compared to using a monochromatic beam.^[^ ^76^ ^]^ Radioluminescent spectra of doped materials such as LaF_3_:Ce mostly depend on the activator ions that act as emission centers, here Ce^3+^. Modifying the excitation energy only alters the spatial distribution of the charges generated in the material after the absorption of the primary ionizing radiation. After various energy transfer steps, a fraction of these charges reaches the emission centers and induces radioluminescence emission. Therefore, tuning the X‐ray energy may affect the light yield as well as the timing properties of the materials but not the emission spectra determined by the activator itself.

##### Geant4 Simulations

Geant4 (version 4.10.6, patch 01), a free toolkit released by an international consortium,^[^ ^54^ ^]^ was used to generate the spectra of secondary electrons and photons generated when X‐ray photons interact with either LaF_3_:Ce or water. The Livermore low energy package was used as it is the most suitable package to simulate low energy interactions with inorganic materials. The geometry was identical as we previously described.^[^ ^29^ ^]^ Briefly, a rod‐like structure of LaF_3_:Ce (1 nm^2^ area and 1 mm long) was placed in the middle of an empty sphere representing the “Sensitive Detector,” able to histogram the secondary particles. The LaF_3_:Ce rod was subjected to a monochromatic X‐ray radiation that interacts in the center of the 1 nm^2^ surface. The energy of the X‐rays was tuned at 30 keV, 50 keV and 80 keV. For each condition, the LaF_3_:Ce rod was irradiated by a minimum of 1.10^6^ photons. As a control, the LaF_3_:Ce rod was replaced by a similar rod composed of water (1 nm^2^ area and 1 mm long) that represents tissues.

##### Cell Culture and Reagents

Glioblastoma cell lines U‐87 MG (human) and F98 (rat) were obtained from the American Type Culture Collection (ATCC). The two cell lines were maintained in Dulbecco's modified eagle's medium (DMEM, high glucose, Glutamax, ThermoFisher) supplemented with 10% (v/v) fetal bovine serum and 1% (v/v) penicillin/streptomycin. The cells were maintained at standard culture conditions (37 °C, 5% CO_2_) and were typically passaged twice a week with a ratio of 1:8 and 1:10 for U‐87 MG and F98, respectively.

Tumor spheroids were formed by seeding cells in ultra‐low adhesion 96‐well plates (Corning). Cells were plated at a density of 5000 cells per well by adding 100 µL of a 50000 cells.mL^−1^ suspension in each well. Spheroids were typically formed within a few hours. Spheroids were imaged daily using a Zeiss AxioObserver Z1 videomicroscope at 10X magnification. Spheroid area and growth were derived from the bright‐field images using a customized Matlab code that binarizes images using an adaptive thresholding allowing to outline the spheroid.

In all experiments, nanoparticles were added to the spheroid culture medium 24 h after culture initiation. Nanoparticle suspensions were prepared with twice the desired concentration in full medium, and 100 µL of this 2X‐concentration suspension was added in the wells that already contained 100 µL of medium, in order to reach the desired concentration.

##### Viability and Necrosis Assay

To quantify the viability and the necrotic population, a live/dead protocol that was previously optimized was used.^[^ ^57^, ^77^ ^]^ Two control groups were needed for this assay: a no treatment group and a group where 100% of the cells were necrotic. This second group was referred to as the “total killing (TK)” group. To prepare the TK control group, the spheroids were first fixed for 2 min in 50 µL of 4% paraformaldehyde prepared in PBS (without Ca^2+^, Mg^2+^, Gibco). The 50 µL paraformaldehyde were then replaced by 50 µL of Triton X‐100 to permeabilize the cellular membranes. After 45 min incubation, spheroids were washed twice using 100 µL of 0.1 mol.L^−1^ glycine to remove any remaining traces of Triton X‐100. Finally, the spheroids from the TK groups were left in 100 µL of PBS. The staining solution was then prepared in PBS to reach 4 µm calcein AM and 6 µm propidium iodine (PI). The volume of each well containing a spheroid was brought to 50 µL, to which 50 µL of this live/dead mix was added. After 30 min incubation at standard culture conditions, the spheroids were imaged using a Zeiss LSM 710 confocal laser scanning microscopy at either 5X or 10X objectives (NA 0.3). The live (cleaved calcein AM emission) and dead (PI emission) fluorescent signals were collected at *λ*_exc _= 488 nm/*λ*_em _= 500–540 nm and *λ*_exc _= 560 nm/*λ*_em _= 600–650 nm, respectively. The viability and extend of necrosis were then derived using the CALYPSO methodology as previously described.^[^ ^57^ ^]^

##### Statistical Analysis for In Vitro Experiments

All statistical analyses were performed using Graphpad Prism 8 (La Jolla, CA). For each experiment, the spheroid size and necrosis (PI fluorescence intensity) were normalized to the no treatment controls. Automated outlier removal was then applied (ROUT method, *Q* = 2). All data passed the D'Agostino & Pearson normality test and were thus statistically analyzed using One‐way ANOVA and Sidak's multiple comparisons test. Logistic growth fits were applied to obtain the growth curves. Dose response fits were statistically compared using an extra sum‐of‐squares f‐test. In the figure panels, statistical significance is indicated with single‐ (*p* ≤ 0.05), double‐ (*p* ≤ 0.01), triple‐ (*p* ≤ 0.005), or quadruple asterisks (*p* ≤ 0.001).

##### Spatially Resolved X‐Ray Fluorescence Imaging on Spheroids

The spheroid cultures (F98 or U‐87 MG) were incubated with 0.1 mg mL^−1^ LaF_3_:Ce on culture day 2. After 24 h of incubation, unbound nanoscintillators were washed away: 180 µL was removed from each well and 200 µL of PBS was added and subsequently removed. Spheroids were fixed in 150 µL of 4% paraformaldehyde prepared in OPTIMA water for ultra‐low trace metal analysis (Fisher Scientific, UK). After 20 min of fixation, the spheroids were washed twice with 200 µL of OPTIMA water. The spheroids were harvested from the well using a large orifice pipette tip and deposited on a glass coverslip. The spheroids were then glued to 200 µm diameter quartz capillaries under a microscope and kept under ambient conditions for several days before imaging. The X‐ray fluorescence tomography imaging was performed on the P06 beamline of the German synchrotron PETRA III, Hamburg. The spheroids were imaged using a 1 × 1 µm^2^ beam spot with an energy tuned at 14 keV and the X‐ray fluorescence was recorded using a Maia 384‐C detector mounted in backscattering geometry at the microfocus end‐station of the beamline.^[^ ^78^ ^]^ In order to reconstruct the final 3D image of the spheroids, 120 projections were acquired over a rotation between 0° and 360°. Tomographic reconstruction was performed using a maximum‐likelihood expectation‐maximization algorithm.^[^ ^79^ ^]^ The resolution was 2 µm.pixel^−1^ and the acquisition time was fixed at 2 ms.pixel^−1^. The general structure of the spheroid was reconstructed using the prolific zinc present in cells; the amount of lanthanum was quantitatively measured using the tools included in the GeoPIXE software package.^[^ ^80^ ^]^

##### Assessment of the LaF_3_:Ce Toxicity In Vivo

All animal experiments were performed in accordance with national legislation, and with the approval of the institutional and national (“Ministère de l’Enseignement Supérieure et de la Recherche") animal ethics committees (APAFIS #2016060114324507). A dose‐escalation study was performed to identify the highest concentrations of LaF_3_:Ce nanoparticles in suspension that can be safely administered to healthy rats by either CED or iv injections. Seven groups of healthy Fischer rats of approximately 6 weeks‐old (average weight: 208 g) were randomly formed as described in **Table** **1**. As this concerned an exploratory study, a minimal sample size of *N* = 3 rats/group was included. Before injection, the anesthesia was initiated using 4% isoflurane and maintained via intraperitoneal injection of a ketamine (80 mg kg^−1^ bw)/xylazine (10 mg kg^−1^ bw) cocktail prepared in sterile water. An ophthalmic ointment (Ocry‐gel 10 g, TVM‐lab) was applied in each eye to prevent corneal dehydration during the surgical procedure. The iv injections were performed via the tail vein using an iv catheter (Becton Dickinson Insyte‐N 24G yellow). For the CED injection, the surgery was performed using a stereotactic headframe (David Kopf instruments, Tujunga, California) on which a heating pad was installed to prevent body temperature loss. The nanoparticles were injected into the right caudate nucleus using a Hamilton syringe (1702 RN, 32/25 mm/4): A skin incision was made, the skull was exposed and a burr hole was drilled 3.5 mm to the right of the bregma. The needle was first taken down to 6 mm deep and withdrawn from 0.5 mm, so that the injection would be performed at 5.5 mm deep. 20 µL of a suspension of increasing concentration of LaF_3_:Ce in PBS were injected at a constant speed of 1 µL.min^−1^ controlled by a syringe pump (KDS310; Geneq, Inc., Montréal, Quebec, Canada). After the injection was completed, the needle was left in place for 1 min before being slowly removed. The hole in the skull was filled with bone wax, the surgical field was cleaned using a povidone‐iodine solution and the scalp was sutured. The concentration of LaF_3_:Ce nanoparticles was doubled every day unless a toxic reaction was observed in the previous groups. All the animals were observed and weighted daily after the injection. For each group, blood samples (500 µL) were collected 30 min, 7, and 14 days after the injection was completed, under isoflurane anesthesia; the anesthesia was initiated with 4% isoflurane in an induction chamber and maintained with 1.5% isoflurane delivered by a mask. The blood samples (500 µL) were collected in 2 mL Eppendorf tubes through an iv catheter (Becton Dickinson Insyte‐N 24G yellow) placed in the tail vein; both the catheter and the tube were rinsed with heparin before use.

**Table 1:** Experimental conditions used for the seven treated groups

Group	Mode of administration	LaF_3_:Ce Concentration [mg mL^−1^]	Volume injected [µL]	Rate [µL.min^−1^]
1	CED	0	20	1
2	CED	25	20	1
3	iv	25	1000	200
4	CED	50	20	1
5	iv	50	1000	200
6	CED	100	20	1
7	iv	35	1000	200

##### Quantification of the Hepato‐, Nephro‐, and Cardiac Toxicity

Between collection and processing, the samples were kept on ice. They were centrifuged for 5 min at 10000 rpm to separate the plasma from the blood cells; the plasma (supernatant) was transferred into a 500 µL Eppendorf tube and stored at −80 °C. The plasma samples were analyzed using a M‐Scan II (Melet‐Schloesing Laboratories) and the VET‐16 reagent rotors, with which plasma concentrations of aspartate transaminase and alanine transaminase , creatinine and urea, and creatine kinase were determined as markers of hepato‐, nephro‐, and cardiac/muscle toxicity, respectively.

##### Orthotopic Tumor Inoculation through Stereotaxic Surgery

Approximately 6 weeks old male Fischer rats (average weight: 233 g) were purchased from Charles River Laboratory (L'Arbresles, Rhône, France). The injection of F98 glioblastoma cells was performed using the anesthesia protocol described in the section “Assessment of the LaF_3_:Ce toxicity in vivo.” The surgery was performed using a stereotactic headframe (David Kopf instruments, Tujunga, California) on which a heating pad was installed to prevent body temperature loss. The F98 rat glioma cells were injected into the right caudate nucleus using a Hamilton syringe (1702N‐32G‐25 mm). A skin incision was made, the skull was exposed and a burr hole was drilled 3.5 mm to the right of the bregma.^[^ ^61^, ^67^, ^81^, ^82^, ^83^ ^]^ The needle was first inserted to a depth of 6 mm below the skull surface and then withdrawn from 0.5 mm before injecting the cells. 1000 cells (5 µL of a 200000 cells.mL^−1^ suspension in DMEM) were stereotactically injected at a controlled speed of 1 µL.min^−1^ using a syringe pump (KDS310; Geneq, Inc., Montréal, Quebec, Canada). After tumor cell implantation, the needle was left in place for 1 min and then slowly withdrawn. The hole left in the skull was filled with bone wax and the surgical field was cleaned with a povidone‐iodine solution before the scalp was sutured. After surgery, the rats were housed in a controlled environment at 21 °C with a day/night cycle of 12 h, no more than three rats per cage; they all had access ad libitum to water and standard laboratory food. Procedures related to animal care conformed to the Guidelines of the French Government.

##### Convection Enhanced Delivery (CED) of LaF_3_:Ce Nanoparticles in Tumor Bearing Rats

Fourteen days after tumor implantation, 20 µL of a suspension of 50 mg mL^−1^ LaF_3_:Ce in PBS were injected intratumorally at the exact same stereotactic coordinates used for previous intracerebral tumor implantations. The anesthesia procedure was identical to the one used for tumor implantation and previously described. The ophthalmic ointment was applied and the animals were placed on the stereotactic headframe over a heating pad. The scalp incision was reopened and the bone wax was removed with a needle. The nanoparticles were injected using a Hamilton syringe (1702 RN, 32/25 mm/4) at a constant speed of 1 µL.min^−1^ controlled by a syringe pump (KDS310; Geneq, Inc., Montréal, Quebec, Canada). After the injection was completed, the needle was left in place for 1 min before being slowly removed. The hole in the skull was filled with bone wax, the surgical field was cleaned using a povidone‐iodine solution, and the scalp was sutured.

##### Pharmacokinetics of the LaF_3_:Ce Nanoparticles in Glioblastoma‐Bearing Rats

LaF_3_:Ce nanoparticles were injected 14 days after tumor implantation through iv injection in the tail vein (*N* = 8 animals, 1 mL of 35 mg mL^−1^ suspension in PBS injected at a rate of approximately 200 µL.min^−1^) or through CED infusion (*N* = 8 animals, 20 µL of 50 mg mL^−1^ suspension in PBS injected at a pace of 1 µL.min^−1^). As a control, four rats received an injection of 20 µL of the vehicle (PBS) through CED injection. 30 min post‐injection, four animals that received an iv injection, four animals that received a CED injection of nanoparticles, as well as two animals that received a CED injection of the vehicle were euthanized and their organs (brain, liver, kidneys, and spleen) were collected. The brains were first frozen by immersion in cold liquid isopentane. All the organs were stored at −80 °C for inductively coupled plasma mass spectrometry (ICP‐MS) analysis. The remaining animals were euthanized 24 h post injection and their organs were collected, stored and analyzed in the same way. As this concerned an exploratory study, a minimal sample size of three rats/group was included. Given the fact that this experiment concerned diseased animals (i.e., carrying orthotopic glioblastoma), it was anticipated that the repeated anesthesia could prove lethal to some animals and thus included an extra animal in each group. Throughout the study, no animals died as a result of the experimental procedures, and the depicted data were thus obtained from a total of *N* = 4 rats/group.

##### ICP‐MS Analysis to Quantify La in the Organs

Before analysis, the organs were weighted and digested. Briefly, the organs were first dissolved by immersion in a typical volume of 5 to 8 mL of nitric acid in closed perfluoro‐alkoxy tubing kept in a cold environment for 5 days. They were then placed on a heating plate and maintained at 95 °C for 12 h until the content of the tube became completely clear. The samples were then diluted and their content in lanthanum was subsequently measured using a Triple Quadrupole ICP‐MS.

##### Tumor Imaging Using MRI

MR imaging was performed at the IRMaGe platform from the Grenoble Institute of Neurosciences using a 4.7‐Tesla MRI machine (Avance III console; Bruker). The procedures were performed under isoflurane anesthesia induced in a chamber with 4% isoflurane and maintained with 1.5% isoflurane using masks installed in the MRI magnet. A T2‐weighted sequence was acquired for each animal; this sequence can distinguish the tumor without injecting a contrast agent. The images were acquired 12 days post tumor implantation in order to randomize the animals in each treatment group based on the size of the tumor 2 days before treatment. Thus, each group contained equivalent numbers of animals bearing large and small tumors.

##### CT Imaging and Data Analysis of LaF_3_:Ce Nanoparticles Distribution

The tomography images were acquired using a high‐purity Germanium detector (Eurisys Mesures, Lingolsheim, France) with 0.35 mm pixel size, installed on the medical beamline (ID17) at the European Synchrotron Research Facility (ESRF, Grenoble, France).^[^ ^84^ ^]^ Upon ketamine/xylazine anesthesia delivered using the same protocol described for the tumor inoculation, the animals were installed in a vertical position on a stereotactic frame and imaged when rotating. Coronal CT scans (1‐mm slice thickness, 1‐mm spacing) were acquired before completing the radiotherapy treatment, that is, 30 min after LaF_3_:Ce injection as well as at various time points after injection. Image reconstruction was performed using the SNARK filtered back projection algorithm.^[^ ^85^ ^]^ The lanthanum concentrations in all voxels of the rat brain were calculated as we previously described,^[^ ^86^ ^]^ using (*μ*/*ρ*)_La_ = 14.47 cm^2^.g^−1^ and (*μ*/*ρ*)_ICRU4 Tissue_ = 0.226 cm^2^.g^−1^ at 50 keV.^[^ ^53^ ^]^

##### Monochromatic Synchrotron Radiation Therapy

For both in vitro and in vivo experiments, radiation therapy was delivered by a monochromatic X‐ray beam on the medical beamline (ID17) at the ESRF. X‐rays of three different energies (30, 50, and 80 keV) were used for in vitro experiments, whereas the beam energy was fixed at 50 keV for the preclinical study. In vitro irradiations were delivered from a single incidence using a 100 mm‐wide, 1 mm‐high beam. The plate containing the cells was inclined 30° relative to the incident X‐ray beam. The plate was scanned vertically through the X‐ray beam to irradiate a total height of 8 cm. The X‐ray dose rate was measured using an ion chamber (UNIDOS PTW 31 002, Freiburg, Germany) and the number of scans required to deliver the prescribe dose was calculated.

For in vivo experiments, the radiotherapy was delivered stereotactically. The animals that were still anesthetized (ketamine/xylazine) from the injection of the nanoparticles were set on a stereotactic frame, in a vertical position. A single 15 Gy dose was delivered to the tumor location 14 days after implantation, using monochromatic 50 keV X‐rays (50‐eV energy bandwidth).^[^ ^84^ ^]^ CT of the rat head was first acquired, as described in the previous subsection, to locate the right cerebral hemisphere. The targeted volume was centered on the rotation axis of the irradiation system, and the beam was shaped to 10 mm‐width and 1 mm‐height. The X‐ray dose rate was measured using an ion chamber (UNIDOS PTW 31 002, Freiburg, Germany) and the number of scans required to deliver the total dose to the tumor was calculated assuming that the tumor is surrounded by 1.5 cm of equivalent tissue. Half of the radiation dose was delivered along a first incidence by scanning the animal vertically through the X‐ray beam. The rat was then rotated by 90° to receive the second half of the treatment. The final irradiated target volume encompassed a cube of 10 × 10 × 10 mm^3^.

##### Euthanasia and Organ Collection

All the animals were euthanized by an intracardiac injection of dolethal (Vétoquinol) performed under 4% isoflurane anesthesia. After euthanasia, the organs were collected (brain, liver, kidneys, and spleen depending on the experiments). Livers, kidneys, and spleens were frozen at −80 °C, whereas the brains were frozen by immersion in cold liquid isopentane and then stored at −80 °C.

##### LIBS Imaging

LIBS elemental imaging (ILM Lyon and Ablatom SAS, France) was performed on cryosections analyzed at room temperature, using a setup previously described.^[^ ^87^ ^]^ The optical‐imaging system was equipped with a laser injection line and a 3D motorized platform for sample positioning. The laser used was a Nd:YAG 1064 nm with 8 ns pulses and a frequency of 100 Hz. The measurements were performed under 1.5 L.min^−1^ argon gas with accurate control of the focal distance between the objective and the sample. Images were acquired with a 25 µm resolution, using single shot mode. The light emitted by the plasma was guided by optical fibers through two successive Czerny‐Turner spectrometers (600 lines.mm^−1^ grating blazed at 412 nm and 1800 lines.mm^−1^ grating blazed at 213 nm) and was finally collected by an intensified charge‐coupled device (I‐CCD) camera (Shamrock 505 and Shamrock 303, Andor Technology). The I‐CCD camera was synchronized with the laser and the spectrum was acquired with a delay of 700 ns after the laser pulse, a gate of 5 µs and a gain of 1500. The widths of the entrance slit of the two spectrometers were set to 35 µm and 50 µm, respectively, leading to a spectral resolution of 0.15 nm. The acquisition and data analysis were performed using a custom‐developed LabVIEW software.

## Conflict of Interest

The authors declare no conflict of interest.

## Supporting information

Supporting Information
