# Supplementary material for: Radiation Dose‐Enhancement Is a Potent Radiotherapeutic Effect of Rare‐Earth Composite Nanoscintillators in Preclinical Models of Glioblastoma
Source: Adv Sci (Weinh). 2020 Sep 7;7(20):2001675. doi: 10.1002/advs.202001675 (PMC7578894; doi:10.1002/advs.202001675)
Supplement: Supplementary file 1 — Supporting Information [file ADVS-7-2001675-s001.pdf]

© 2020 Wiley-VCH GmbH

ADVANCED SCIENCE Open Access

Supporting Information

for *Adv. Sci.,* DOI: 10.1002/ advs.202001675

Radiation Dose-Enhancement Is a Potent Radiotherapeutic Effect of Rare-Earth Composite Nanoscintillators in Preclinical Models of Glioblastoma

Anne-Laure Bulin, Mans Broekgaarden, Frédéric Chaput, Victor Baisamy, Jan Garrevoet, Benoît Busser, Dennis Brueckner, Antonia Youssef, Jean-Luc Ravanat, Christophe Dujardin, Vincent Motto-Ros, Frédéric Lerouge, Sylvain Bohic, Lucie Sancey, and Hélène Elleaume**

© 2020 Wiley-VCH GmbH

Supporting Information

Radiation dose-enhancement is a potent radiotherapeutic effect of rare-earth composite nanoscintillators in preclinical models of glioblastoma

Anne-Laure Bulin, Mans Broekgaarden, Frédéric Chaput, Victor Baisamy, Jan Garrevoet, Benoît Busser, Dennis Brueckner, Antonia Youssef, Jean-Luc Ravanat, Christophe Dujardin, Vincent Motto-Ros, Frédéric Lerouge, Sylvain Bohic, Lucie Sancey and Hélène Elleaume**

SI 1: Interpretation of the Fourier Transform Infrared-Attenuated Total Reflectance spectra.
The synthesized nanoparticles present the remaining pyrrolidinone on their surface through coordination to rare earth ions. The first proof of coordination is highlighted by the band at 1648 cm^{-1} corresponding to the n(C=O) stretching vibration. The band observed at 1600 cm^{-1} is characteristic of N-C=O vibrations in secondary amides, suggesting that nitrogen atoms are also involved in the interactions with rare earth atoms located at the surface of the particles. Finally, presence of the organic molecules on the particles is characterized with C-H deformations observed at 1400 cm^{-1} and the bands in the range 2700-2950 cm^{-1}. After functionalization with phosphate molecules, we observe the characteristic bands of the adsorbed residual water molecules (3400 cm^{-1} and 1635 cm^{-1}), as well as the signature of the phosphate molecules in interaction with the surface of the nanoparticles (900 cm^{-1} and 1100 cm^{-1}).

SI 2: Interpretation of the differences observed between the photo- and the radio-luminescence spectra.
Because different light emission mechanisms are involved when optical or ionizing radiation excitations are involved, the photo- and radio-luminescence spectra differ and the relative intensity emitted by regular (310 nm) and perturbed (340 nm) Ce sites varies. Under optical excitation, this relative intensity is driven by the relative optical absorption of both sites, their abundance and their relative quantum efficiencies. Under ionizing radiations, the optical absorption is not involved and the sequential carrier trapping is considered as the dominant mechanism. This mechanism is driven by the Ce content as well as the concentration of traps. Close to the surface and consequently in nanoparticles, it is admitted that the concentration of trap carrier is higher, and therefore lowers the scintillation efficiency.[88]

SI 3: Identification of the optimal X-ray energy to induce a radiation dose-enhancement effect and spectra of the secondary photons produced upon X-ray irradiation of LaF_3:Ce and water.
To identify the optimal X-ray energy for radiation dose-enhancement, two factors need to be considered: 1) the predominance of the photoelectric effect as this will maximize the radiation

dose-enhancement effect, and 2) the relative absorption of the high-Z element regarding water/soft tissues. For the first factor, the simulations demonstrated that photoelectric effect largely predominates (>95%) in LaF_3:Ce for all three excitation energies (30, 50 and 80 keV). However, these proportions only indicate how the interaction happens regardless of the probability of the interaction happening. This probability is the second factor and is described by the dose-enhancement factor (DEF). The DEF is defined as the ratio of the dose deposited in presence of the high Z-element relative to the dose in water/soft tissues. In a first approximation, assuming electronic equilibrium, the DEF can be estimated as DEF= $(\mu_{en}/\rho)_{E,Z}/(\mu_{en}/\rho)_{E,water}$, where E is the energy of the X-ray photons. This ratio is shown Figure 2.B and is maximum at 50 keV (DEF=107.2) compared to 80 keV (DEF=70.7) and 30 keV (DEF=42.2).

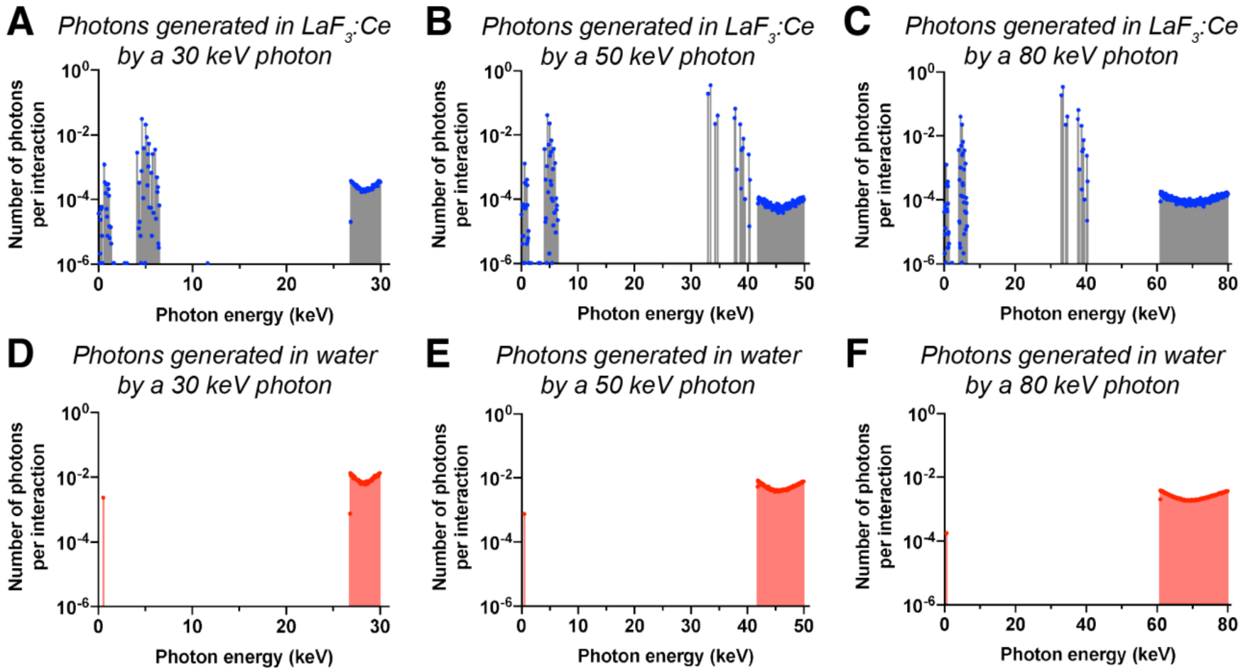

Figure S1. Photons generated in LaF_3:Ce and water (i.e. tissues) after interaction with an orthovoltage X-ray. A-C) Spectra of the photons generated in LaF_3:Ce after interaction with a X-ray photon of 30 keV (A), 50 keV (B) or 80 keV (C). **D-F)** Spectra of the photons generated in water after interaction with a X-ray photon of 30 keV (D), 50 keV (E) or 80 keV (F).

SI 4: Toxicity and uptake of LaF_3:Ce nanoscintillators by U-87 MG microtumors

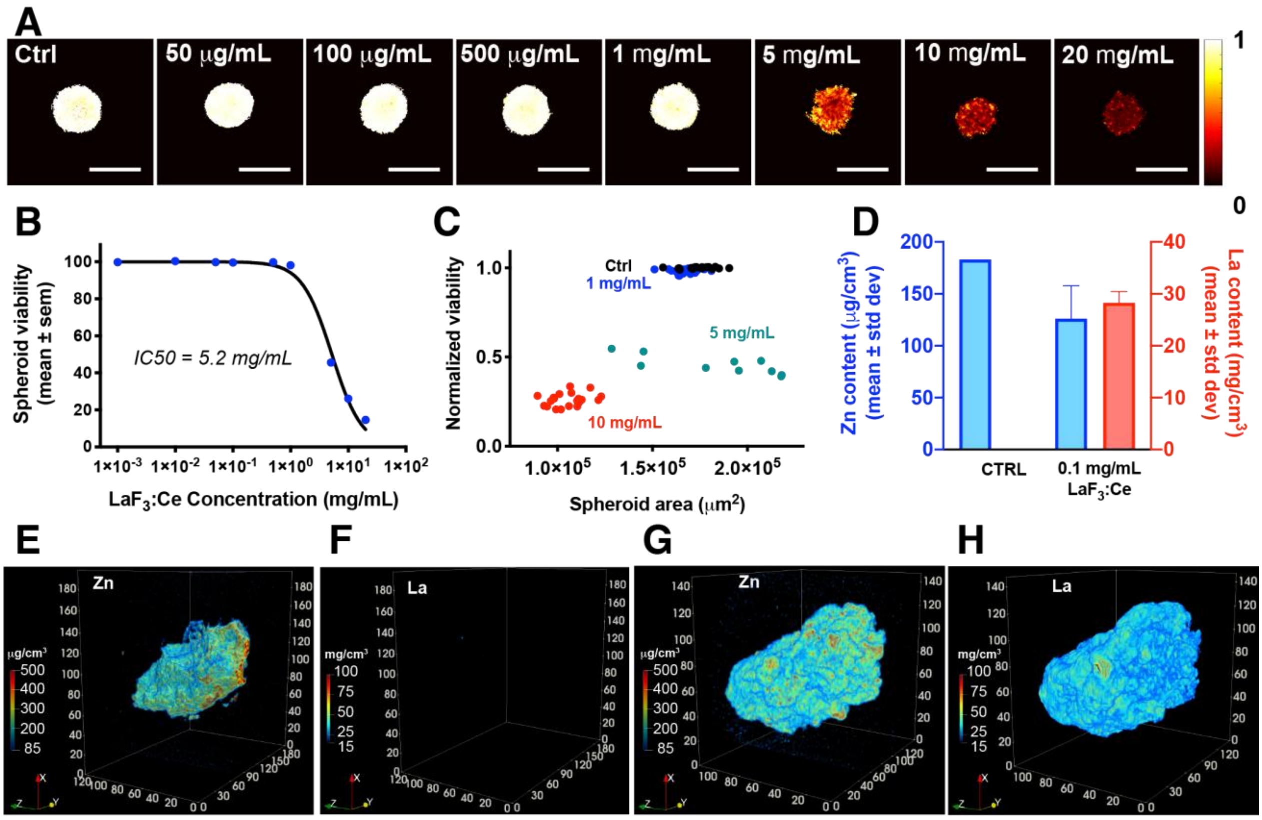

Figure S2. LaF_3:Ce strongly accumulate and distribute well within U-87 MG models of glioblastoma at non-toxic concentration. A) Viability heatmaps of U-87 MG spheroids subjected to increasing concentration of LaF_3:Ce nanoscintillators for 24 hours. White pixels indicate a maximal viability, whereas black pixels depict minimal viability. Scale bar = 500 µm. **B)** Quantified viability of U-87 MG spheroids measured after 24 hours of incubation with increasing concentrations of LaF_3:Ce. Data represents average ± standard errors, N=7-25 spheroids/group from 2 technical repeats. **C)** Normalized viability plotted as a function of the spheroid area for selected LaF_3:Ce concentrations. Each data point represents a single spheroid. **D)** Quantification of the amount of Zn (physiological element present in the cells) and La contained in the spheroids by analyses of the images acquired by X-ray fluorescence microscopy. Data represents mean ± standard deviation. **E-H)** Images acquired using X-ray fluorescence microscopy on a spheroid that was not subjected to nanoparticle (E, F) and to a spheroid that was exposed to 0.1 $mg.mL^{-1}$ LaF_3:Ce for 24 hours. (G, H). E, G) Zn concentration. F, H) La concentration. The x, y and z axis are expressed in µm.

SI 5: Radiotherapeutic effect of the LaF_3:Ce nanoscintillators on U-87 MG microtumor cultures

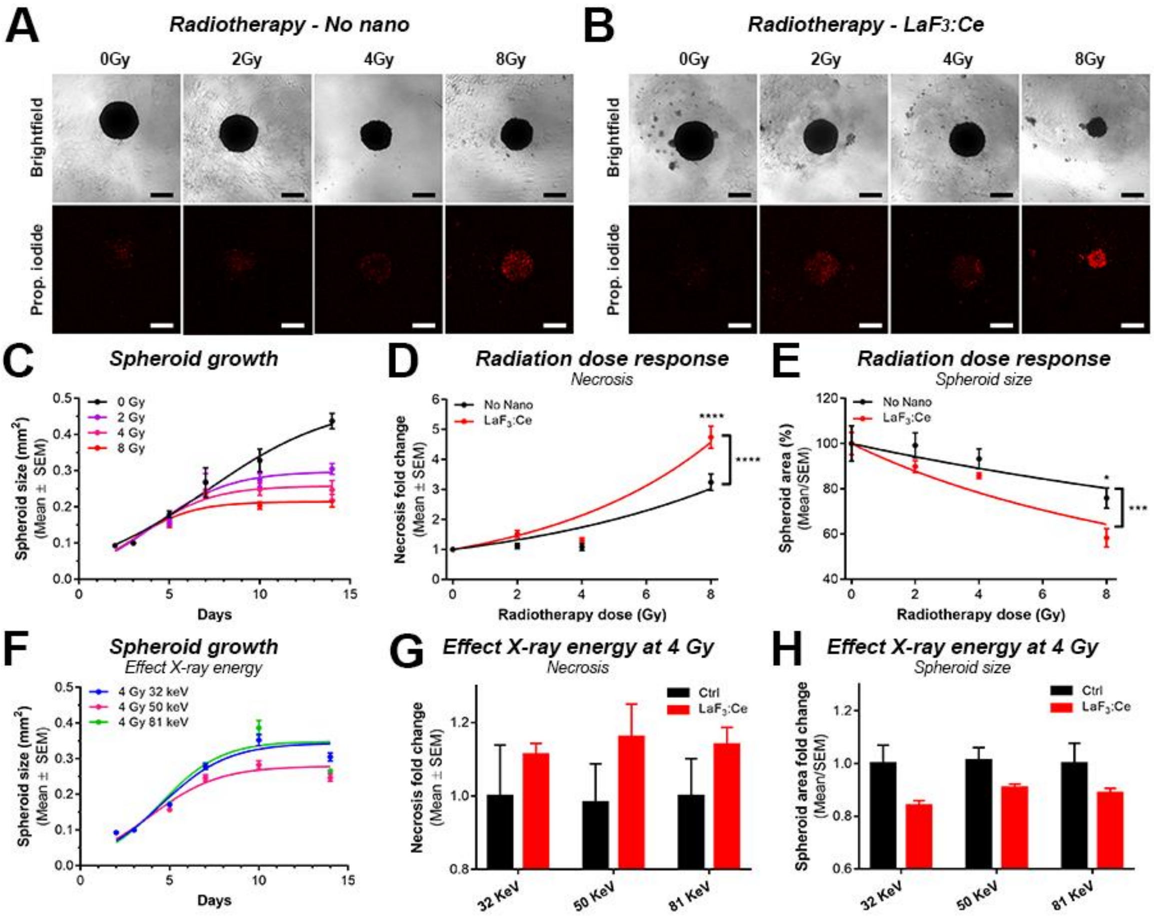

Figure S3. LaF_3:Ce nanoscintillators induce a radiation dose-enhancement effect *in vitro* on U-87 MG spheroids. A-B) Confocal fluorescence microscopy images of U-87 MG spheroids taken on culture day 10, i.e. 7 days following radiotherapy delivered in absence of nanoscintillator (A), or after a 24 hours incubation with 0.1 mg.mL^{-1} nanoscintillators (B). Brightfield and propidium iodide emission (necrosis) images of representative spheroids of each treatment condition are represented. Scale bar = 100 µm. **C)** Logistic growth curves of U-87 MG spheroids treated by synchrotron radiation therapy at 50 keV with a dose of 0 Gy (black), 2 Gy (purple), 4 Gy (pink), or 8 Gy (red), without nanoparticle. **D)** Radiotherapy dose-response fitted as a function of spheroid necrosis. Data was normalized to the 0 Gy controls of each group, and fitted with a dose-response fit. At each dose, the data from both groups was statistically compared with a one-way ANOVA and Sidak's multiple comparisons test. **E)** Radiotherapy dose-response fitted as a function of normalized spheroid size. Data was normalized to the 0 Gy controls of each group, and fitted with a dose-response fit. At each dose, the data from both groups was statistically compared with a one-way ANOVA and Sidak's multiple comparisons test. **F)** Logistic growth curves of U-87 MG spheroids treated with synchrotron radiation therapy at a dose of 4 Gy, delivered at either 30 keV (blue), 50 keV (red), or 80 keV (green) X-ray energies (no nanoparticles). **G)** Analysis of spheroid necrosis, and **H)** analysis of normalized spheroid size following radiotherapy at photon energies of either 30 keV, 50 keV, or 80 keV. Data was plotted as a fold-change compared to the "No nano" control groups subjected to the same radiotherapy dose. In all panels, the depicted data was obtained from N=12-24, and statistical significance is indicated as "*" ($p < 0.05$), "**" ($p < 0.01$), "***" ($p < 0.005$), or "****" ($p < 0.001$).

SI 6: LaF_3:Ce nanoscintillators can be safely administered to healthy rats

A concentration of 25 mg.mL^{-1} was well tolerated by all the animals when injected either through intracerebral CED (20 µL, i.e. 2.1 mg.kg^{-1} bw) or iv (1 mL, i.e. 107.3 mg.kg^{-1} bw). We then escalated up to 50 mg.mL^{-1} (4.3 mg.kg^{-1} bw for CED and 214.6 mg.kg^{-1} bw for iv). While this concentration was well tolerated when injected by CED, it appeared to induce an acute and lethal toxicity when injected intravenously. Two animals in the 50 mg.mL^{-1} iv injection group did not survive the treatment. Therefore, for the last groups of animals we increased the concentration up to 100 mg.mL^{-1} for the CED (8.6 mg.kg^{-1} bw) and reduced the iv dose down to 35 mg.mL^{-1} (150.2 mg.kg^{-1} bw) (Figure 5 and S4), which were well-tolerated.

Iv administration of LaF_3:Ce caused weight loss for all groups one day after injection, but the animals started to regain weight at the same speed as the control group (Figure 5.A). Based on animal weight and behavior, intracerebral CED of LaF_3:Ce was well-tolerated up to 50 mg.mL^{-1}. At 100 mg.mL^{-1}, there was a notable weight loss of almost 10% within the first three days following the injection from which the animals recovered at day 4 and onwards (Figure 5.B). Fourteen days after the injection, all the animals were euthanized and their organs were collected. There was no accumulation of LaF_3:Ce nanoparticles in the kidney, liver or spleen after CED, yet iv injection induced kidney retention for concentrations higher than 35 mg.mL^{-1} and liver and spleen retention for suspensions with concentrations of 25 mg.mL^{-1} (Figure 5.C-E). However, the accumulation in the different organs corresponds to only a small fraction of the total injected amount (Figure S4.B). Because of the small size of the nanoparticles (< 10 nm), renal excretion may be involved.[89] However, as long-term retention was observed in the liver and the spleen of the animal after iv injection, regardless of the concentrations, the hepatobiliary elimination route remains the most probable elimination track for these nanoparticles.

Evaluation of plasma biomarkers for hepato-, nephro- and cardiac/muscle toxicity at various timepoints after injection of the LaF_3:Ce nanoscintillators shows variations contained within a

range that indicates no toxicity (Figure 5.F-J). Minor, transient and dose-dependent oscillations are observed 24 hours after CED and iv injection. However, the ratios return to baseline before day 8, demonstrating a lack of hepato- and nephro-toxicity. Lastly, CED caused no notable elevations in creatine kinase levels, whereas an acute elevation was observed for iv injections (Figure 5.J). No prolonged elevations were observed in any of the treatment groups.

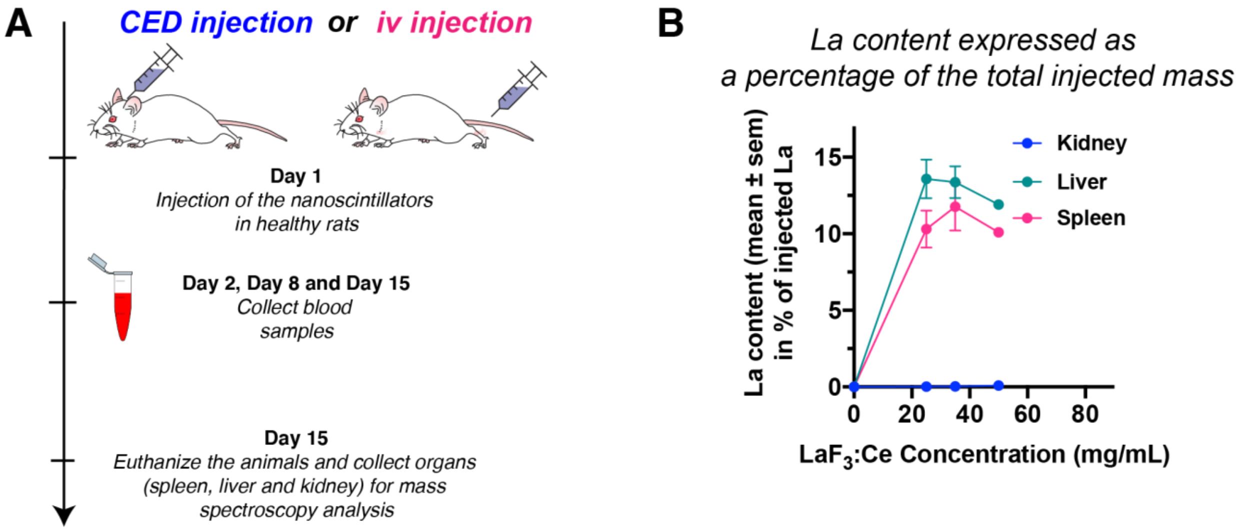

Figure S4. LaF_3:Ce nanoscintillators can be safely administered to healthy rats. **A)** Experimental outline: LaF_3:Ce nanoparticles are injected on day 1 by intravenous (iv) or convection-enhanced delivery (CED) to healthy rats. 24 hours, 7- and 14-days post-injection, blood samples are collected to investigate nephrotoxicity, hepatotoxicity and cardiac/muscle toxicity. On day 15, the animals are euthanized and their organs (kidney, liver and spleen) are collected to quantify the amount of La accumulated using inductively coupled plasma mass spectrometry (ICP-MS). **B)** La content, presented as a percentage of the total injected amount, measured in the kidney, liver and spleen after iv injection of increasing concentrations of LaF_3:Ce.

SI 7: Persistence of the LaF_3:Ce after CED injection and biodistribution of the nanoscintillators after iv and CED injection

Computed tomography (CT) revealed that iv injection did not lead to detectable concentrations of LaF_3:Ce in the tumor tissue. Contrariwise, LaF_3:Ce injected by CED created a strong contrast on CT images 30 min post CED injection (Figure 6.B), which is approximately 1 mg.mL^{-1}. Contrariwise, when injected by CED, the nanoparticles create a strong contrast on CT images 30 min post injection (Figure 6.B). Image analysis quantified the local concentration at 7.5 mg.mL^{-1} that remained stable for up to 43 hours (Figure S5.A-B). These findings were

supported by quantification of La in the whole brain with inductively coupled plasma-mass spectrometry (ICP-MS).

Biodistribution following iv injection (Figure 6.C) revealed that LaF_3:Ce accumulated in the brain, reaching a maximum of 0.01 mg (0.04% of the injected dose) at 30 min. This accumulation was attributed to a disrupted tumor blood-brain barrier, as it was previously observed with this tumor model.[90] A strong decrease was measured between 30 min and 24 hours. Iv injection also resulted in LaF_3:Ce retention in the liver (954 ± 241 ppm) and the spleen (2937 ± 337 ppm) as measured after 24 hours (Figure 6.C), corresponding to approximately 12.5% and 12% of the total amount of La injected (Figure S5.C). Combined with our aforementioned data acquired 14 days post iv injection on healthy rats, the liver and the spleen contain an average of 707 ppm and 1427 ppm, respectively (Figure 5.D-E), indicating a slow elimination of the LaF_3:Ce nanoparticles from the liver and the spleen.

When injected by CED, the concentration of La remains stable in the brain over at least 24 hours (Figure 6.D), corroborating the CT measurements. CT images acquired up to 43 hours post-injection (Figure S5.A-B) and laser induced breakdown spectroscopy (LIBS) analysis performed on brains collected after euthanasia up to 106 days post-injection (Figure S6), demonstrate that nanoparticles remain in the brain for a prolonged period of time. However, less than 1 ppm (1 $\mu g.mL^{-1}$) of La was detected in the kidney, liver and spleen at 30 min and 24 hours post CED injection (Figure 6.D).

To facilitate a radiation dose-enhancement effect, tissue concentrations of LaF_3:Ce are required to be in the range of >1 $mg.mL^{-1}$.[35] Based on the CT images, radiation dose-enhancement appears feasible after CED injection, reaching concentrations of 7.5 $mg.mL^{-1}$ at the injection site (Figure 6.E). This was supported by the ICP-MS findings, in which 0.17 $mg.mL^{-1}$ La was detected in the whole brain, a volume of approximately 1600 mm^3. When corrected for the tumor volume of 40 mm^3, as extrapolated from the MRI image performed on day 12 (Figure 6.F), we obtain a tumor concentration of approximately 6.8 $mg.mL^{-1}$ (Figure 6.E). In

contrast, the iv injection of LaF_3:Ce is unlikely to result in sufficient intratumor concentrations of nanoparticles to achieve a radiation dose-enhancement effect. When applying the same calculation for the iv injection, a concentration of 0.20 mg.mL^{-1} LaF_3:Ce in the tumor 30 min after injection is obtained (Figure 6.E).

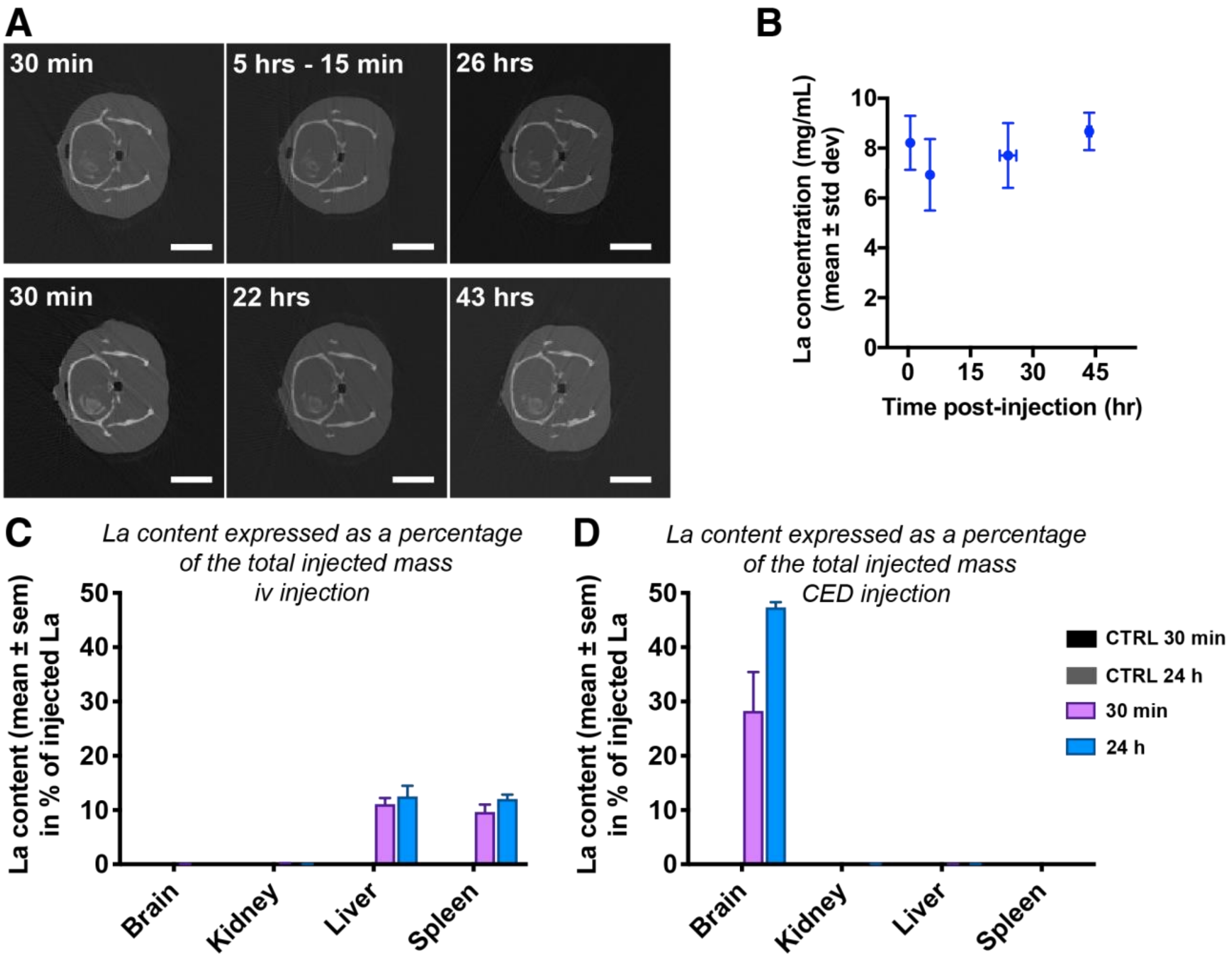

Figure S5. Effect of the injection-to-irradiation delay. A) Computed tomography (CT) images acquired on two different animals, with delays ranging from 30 min to 43 hours after convection-enhanced delivery (CED) injection (20 µL of 50 mg.mL^{-1} LaF_3:Ce). **B)** Concentration of La measured on the CT images as a function of the delay post-injection. Data represents the mean ± standard deviation; N=3/time point. **C-D)** La content measured by inductively coupled plasma mass spectrometry (ICP-MS), plotted as a percentage of the total injected amount, in the brain, kidney, liver and spleen, 30 min and 24 hours post intravenous (iv) (C) or CED (D) injection. N=4 animals/group.

SI 8: La and Ce contained in slices of brain measured by laser induced breakdown spectroscopy

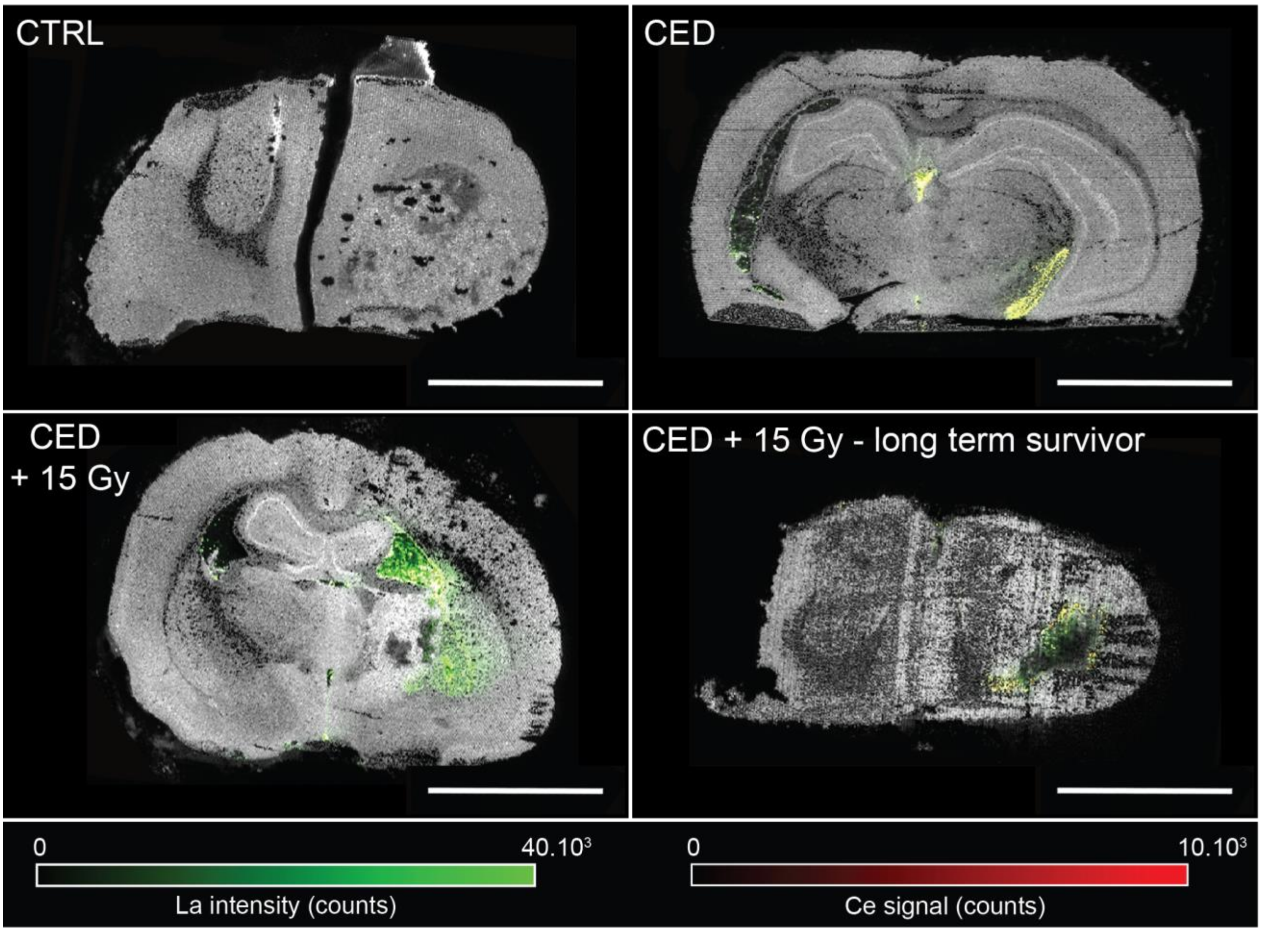

Figure S1. Laser Induced Breakdown Spectroscopy imaging. Images acquired on animals from the control group (euthanized 22 days post implantation), the group that received a convection-enhanced delivery (CED) injection (euthanized 23 days post implantation), the group that received the CED injection + 15 Gy of radiotherapy (euthanized 34 days post implantation) and one of the long-term survivor (CED + 15 Gy) that was euthanized 120 days post implantation without clinical signs. Scale bar = 5 mm.

SI 9: Treatment outcomes after in injection of LaF_3:Ce in rats bearing F98-tumors

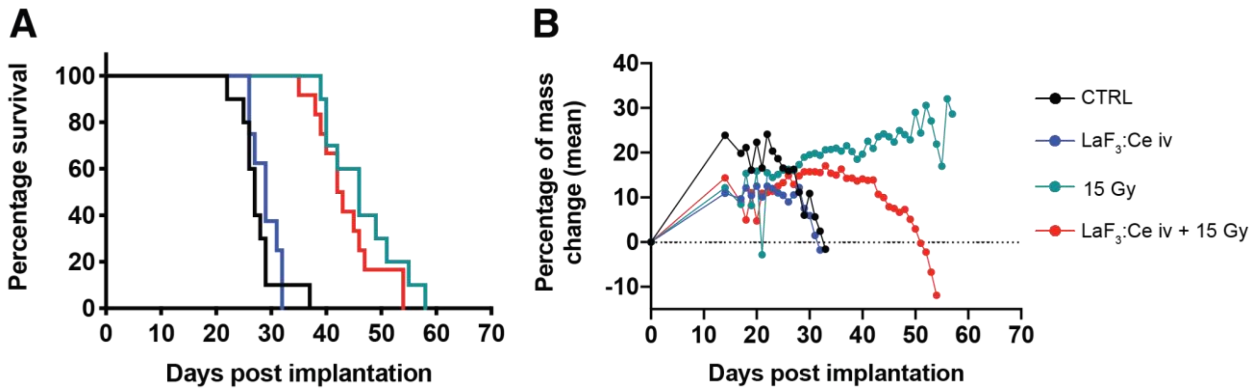

Figure S7. Treatment outcomes after intravenous (iv) injection of LaF_3:Ce nanoparticles. **A)** Survival curves of the rats from the control group compared to the groups that received the iv injection only (1 mL of 35 mg.mL^{-1} LaF_3:Ce), the radiotherapy (15 Gy) and the iv injection (1 mL of 35 mg.mL^{-1} LaF_3:Ce) + radiotherapy (15 Gy). **B)** Mean mass changes of the animals post-implantation from the control group or from the groups that received the iv injection only (1 mL of 35 mg.mL^{-1} LaF_3:Ce), the radiotherapy (15 Gy) or the iv injection (1 mL of 35 mg.mL^{-1} LaF_3:Ce) + radiotherapy (15 Gy).

SI 10: No limiting toxicity of LaF_3:Ce nanoscintillators was observed in diseased animals
Using the survival curves, we distill that the nanoparticles had limited toxicity at the used doses based on two factors. First, the experimental arms where animals received either CED or iv injection of nanoscintillators did not perform worse than the untreated control (Figure 7.A and Figure S7.A). Second, we demonstrated that there was still a high concentration of nanoparticles in the brains of the cured rats, which exhibited no clinical sign of toxicity.
Regarding the intravenous administration of LaF_3:Ce (Figure 7.A), the slightly worse survival may have been caused by two factors. First, the nanoparticles did not accumulate at sufficient levels in the tumor to enhance the radiotherapy outcomes, as demonstrated in Figure 6. Second, the mild systemic toxicity may have had a negative influence on the survival curve of these diseased animals upon exposure to radiotherapy. Nonetheless, it should be emphasized that the survival difference was not statistically significant between the (LaF_3:Ce+15 Gy) and 15 Gy groups.

References

[88] C. Pedrini, B. Moine, J. Gacon, B. Jacquier, *J. Phys. Condens. Matter*. 1992, *4*, 5461.
[89] M. Longmire, P. L. Choyke, H. Kobayashi, *Nanomedicine (Lond)*. 2008, *3*, 703.
[90] B. Lemasson, N. Pannetier, N. Coquery, L. S. B. Boisserand, N. Collomb, N. Schuff, M. Moseley, G. Zaharchuk, E. L. Barbier, T. Christen, *Sci. Rep.* 2016, *6*, 37071.
